# Maternal aging increases offspring adult body size via transmission of donut-shaped mitochondria

**DOI:** 10.1038/s41422-023-00854-8

**Published:** 2023-07-27

**Authors:** Runshuai Zhang, Jinan Fang, Ting Qi, Shihao Zhu, Luxia Yao, Guicun Fang, Yunsheng Li, Xiao Zang, Weina Xu, Wanyu Hao, Shouye Liu, Dan Yang, Di Chen, Jian Yang, Xianjue Ma, Lianfeng Wu

**Affiliations:** 1 Westlake Laboratory of Life Sciences and Biomedicine, Hangzhou, Zhejiang China; 2 School of Life Sciences, Westlake University, Hangzhou, Zhejiang China; 3 Key Laboratory of Growth Regulation and Translational Research of Zhejiang Province, Hangzhou, Zhejiang China; 4 Research Center for Industries of the Future, Westlake University, Hangzhou, Zhejiang China; 5 Institute of Basic Medical Sciences, Westlake Institute for Advanced Study, Hangzhou, Zhejiang China; 6 Microscopy Core Facility, Westlake University, Hangzhou, Zhejiang China; 7 Model Animal Research Center of Medical School, Nanjing University, Nanjing, Jiangsu China; 8 Institute for Molecular Bioscience, University of Queensland, St Lucia, Brisbane, QLD Australia

**Keywords:** Ageing, Energy metabolism

## Abstract

Maternal age at childbearing has continued to increase in recent decades. However, whether and how it influences offspring adult traits are largely unknown. Here, using adult body size as the primary readout, we reveal that maternal rather than paternal age has an evolutionarily conserved effect on offspring adult traits in humans, *Drosophila*, and *Caenorhabditis elegans*. Elucidating the mechanisms of such effects in humans and other long-lived animals remains challenging due to their long life course and difficulties in conducting in vivo studies. We thus employ the short-lived and genetically tractable nematode *C. elegans* to explore the mechanisms underlying the regulation of offspring adult trait by maternal aging. By microscopic analysis, we find that old worms transmit aged mitochondria with a donut-like shape to offspring. These mitochondria are rejuvenated in the offspring’s early life, with their morphology fully restored before adulthood in an AMPK-dependent manner. Mechanistically, we demonstrate that early-life mitochondrial dysfunction activates AMPK, which in turn not only alleviates mitochondrial abnormalities but also activates TGFβ signaling to increase offspring adult size. Together, our findings provide mechanistic insight into the ancient role of maternal aging in shaping the traits of adult offspring.

## Introduction

Over the past decades, there has been a continuing trend for women to delay childbearing decisions until more advanced ages worldwide, possibly due to the pursuit of higher levels of educational and professional attainment.^1^ Thus, interest in studying the effects of maternal age on offspring outcomes, especially early-life survival, and health,^2^ is growing dramatically despite the inconsistent conclusions drawn from studies across species.^1,3,4^ Furthermore, the specific role of the maternal age effect (MAE) in mediating the adult traits of offspring and its underlying mechanism remain poorly understood because sex-specific parental effects are difficult to differentiate^4–6^ and because adult trait development is generally affected by numerous factors during the trajectory from birth to adulthood.

Mitochondria, heritable “powerhouse” organelles, are known to have crucial functions in mediating offspring health and disease, largely through the inheritance of maternal mitochondria.^7,8^ Paternal mitochondria are sequestered by autophagosomes and delivered to lysosomes for degradation upon completion of fertilization.^9,10^ With aging, mitochondria display evident changes in morphology, abundance, and activity.^11^ It has been reported in multiple species that old mothers can pass aged, defective mitochondria to oocytes despite the presence of a tight selection bottleneck in the germline.^12,13^ Recently, aged mitochondria in general have been connected to numerous cellular processes, such as cell fate determination and potential tissue renewal.^14–16^ However, how these aged mitochondria are rejuvenated in organismal offspring remains unexplored, despite the reported involvement of a few signaling pathways in cellular mitochondrial biogenesis, including the mammalian target of rapamycin (mTOR), AMP-activated protein kinase (AMPK), and sirtuin pathways.^11^ The physiological roles that aged mitochondria play in the regulation of offspring adult traits are also largely unknown.

Adult height has been widely used as a model phenotype in human genetics studies,^17,18^ partly because it is a trait that is easy to measure and because it is affected by all the conditions to which an individual is exposed from fertilization to adulthood, including genetic and environmental factors. Classical twin and family studies have consistently estimated that human adult height is ~80% genetically determined,^19,20^ which is supported by data from recent genome-wide association studies.^17,18,21,22^ Apart from the genetic determinants, the remaining factors that account for adult height variation have not been fully explored.

Considering the unsettled effect of parental age on offspring adult traits and its lifelong and multifactorial properties,^4,6,23^ we first analyzed human data from the UK Biobank and revealed that the MAE shapes offspring height and other traits in adulthood. Then, the conserved MAE on offspring adult size regulation was consistently confirmed in both *Drosophila* and *Caenorhabditis elegans* model organisms. In a mechanistic study using *C. elegans*, we observed that aged worms passed donut-shaped mitochondria to their offspring via oocytes. Furthermore, these aged mitochondria epistatically triggered periodic activation of AMPK in the offspring’s early life, which not only stimulated mitochondrial rejuvenation but also triggered the size-increasing process by eliciting the activity of the transforming growth factor beta (TGFβ) signaling pathway. Altogether, our findings identify the evolutionarily conserved role of MAE in programming traits in adult offspring and provide a new mechanistic avenue for studying or targeting the MAE for early-life interventions against disorders in adulthood.

## Results

### MAE is involved in the regulation of offspring adult traits in humans

**Fig. 1:**
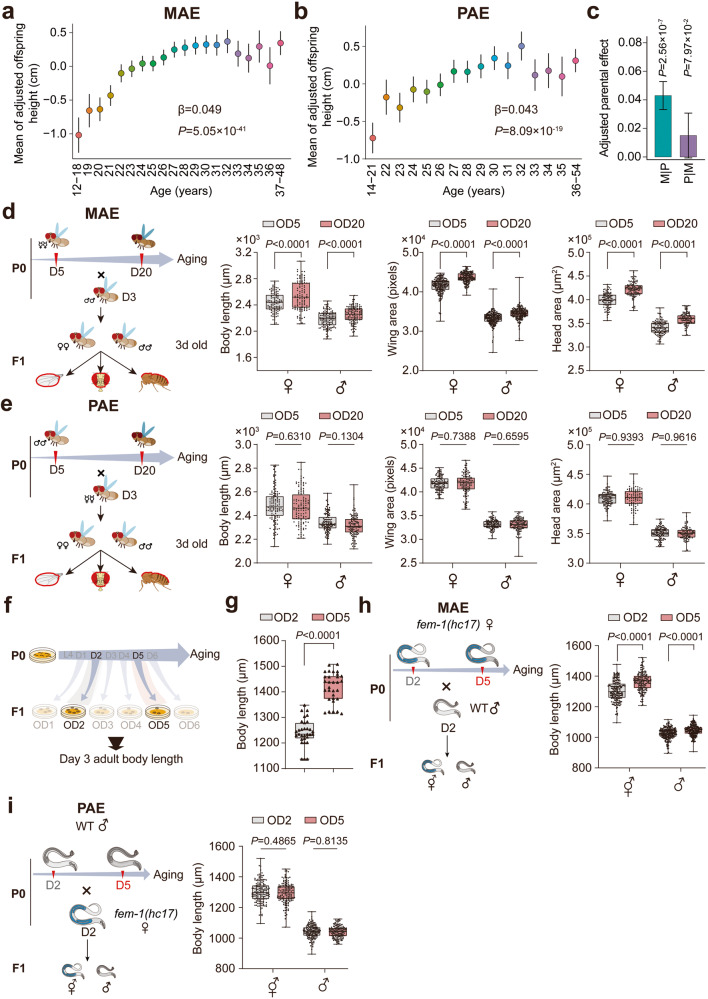
An evolutionally conserved MAE shapes offspring adult traits in humans, *Drosophila* and *C. elegans*. **a**, **b** Relationships of maternal (**a**) and paternal (**b**) age with offspring adult height in humans. **c** Adjusted sex-specific parental age effect on offspring adult height in humans. M | P (P | M) represents the MAE (PAE) when fitted jointly with paternal (maternal) age. **d**, **e** Comparisons of body length, wing size and head area for both male and female offspring from the MAE (**d**) and PAE (**e**) test groups in flies. **f** Strategy for offspring preparation from mothers of different ages (P0) in *C. elegans*. Offspring (F1) born to P0 individuals of Day 1–6 adults (D1–D6) were named OD1–OD6, respectively. All body length measurements in *C. elegans* were conducted on Day 3 of adulthood unless otherwise noted. **g** Adult body length comparison between OD2 and OD5 animals. **h**, **i** Analysis of sex-specific parental age effects on adult body length of offspring from parents of the indicated ages. The experimental designs are shown on the left. The *fem-1(hc17)* mutant animals were employed as females (♀) and crossed with WT males (♂) of the indicated ages to produce offspring for MAE tests.  stands for hermaphrodite. The dots in box plots represent worm or fly numbers, and the triangles in the box pot (**g**) stand for the average adult body lengths of worms in each biological replicate. Box plots in **d**, **e**, **g**–**i**: the centerline is the median, the box range shows the 25th–75th percentiles, and the whiskers indicate the minimum–maximum values. All box plots (**d**, **e**, **g**–**i**) were analyzed by unpaired *t*-test. Biological replicates: 3 (**d**, **e**, **h**, **i**), 26 (**g**).

Our analysis of human data from the UK Biobank showed that both maternal and paternal ages, when analyzed separately, were positively correlated with offspring adult height (Fig. 1a, b; *β* = 0.049, *P* = 5.05 × 10^–41^ for MAE; *β* = 0.043, *P* = 8.09 × 10^–19^ for paternal age effect (PAE)). However, when maternal and paternal ages were fitted jointly in a model (Fig. 1c), the estimate of MAE was almost unchanged and remained highly significant (*β* = 0.047, *P* = 2.56 × 10^–7^), whereas the estimate of PAE decreased substantially and became nonsignificant (*β* = 0.015, *P* = 0.08). These results were consistent with those of the maternal or paternal age-stratified analysis (Supplementary information, Fig. S1a–d), suggesting that the significant PAE shown in the separate analysis was driven by an MAE, potentially due to age-assortative mating. The proportion of variance in offspring adult height explained by the model remained almost unchanged when we fitted a polynomial regression model to account for the nonlinear relationship between maternal age and offspring adult height (Supplementary information, Fig. S2). We further found widespread effects of maternal age on a host of phenotypes, including positive effects on cognitive ability and forced vital capacity and a negative effect on body fat deposit (Supplementary information, Fig. S3).

### Evolutionarily conserved MAE on offspring adult trait in *Drosophila* and *C. elegans*

Next, we employed the fruit fly (*Drosophila melanogaster*), a model organism allowing well-controlled sex and age matchups for parental mating that has been proven useful for studying aging-related transgenerational effects,^24,25^ to explore whether the MAE has an evolutionarily conserved role in mediating offspring adult size (Fig. 1d, e). Twenty-day-old (D20) flies were used as the group of aged mothers, as they have been found to have clear signs of aging, including severe short-term memory loss.^26^ Relative to that of offspring from young fly mothers aged 5 days (OD5), significant increases in overall size, including an increased body length (the sum of the thorax and abdomen) and enlarged wing and head sizes, and body mass were observed in offspring born to D20 flies (OD20) (Fig. 1d; Supplementary information, Fig. S4a–e). The enlarged phenotype was not observed in offspring born to old male flies (Fig. 1e; Supplementary information, Fig. S4a–e), further supporting the evolutionarily conserved role of an MAE, rather than a PAE, in programming the size of adult offspring. We also investigated whether maternal parity would affect the MAE outcomes in flies and found that the MAE-mediated size changes were not influenced by the number of times the mothers had reproduced (Supplementary information, Fig. S4f–i), suggesting that age, rather than the mating behavior per se, is a decisive factor for MAE-mediated size increase.

To further validate the MAE and explore its underlying mechanisms, we chose the nematode *C. elegans*, which has a much shorter lifespan than *D. melanogaster* and is easy to manipulate microscopically and genetically. To rigorously design *C. elegans* experiments for studying the MAE on the adult size of offspring, we collected embryos from mothers on each day of their adulthood until Day 6 (D6), after which sufficient embryos could rarely be collected, and measured the final adult length of offspring on D3 of adulthood, as previously defined^27^ (Fig. 1f). We found that the adult length of offspring increased significantly with parental age from D1 to D6 (Supplementary information, Fig. S5a). D2 mothers are reported to be more mature than D1 mothers in providing yolk to their offspring,^23^ and D5 mothers can produce still larger offspring in terms of adult length than D2 mothers (Fig. 1g). Therefore, we chose D2 mothers as the optimal young mothers and D5 as aged ones for the following phenotypic and mechanistic studies. In the size comparison experiment, we noticed that the sizes of OD5 animals were larger than those of OD2 animals from larval to adulthood stages (Supplementary information, Fig. S5b), indicating that the MAE was initiated in the early-life stages of *C. elegans*. Moreover, a D5 MAE-mediated offspring adult size increase compared to the adult sizes of OD2 animals was consistently detected in 26 independent experiments (Fig. 1g; Supplementary information, Fig. S5c).

Worms are hermaphroditic animals that simultaneously hold oocytes and sperm at reproductive age; hence, sex-specific parental effects are normally difficult to determine. To investigate the parental age effects of specific sexes on offspring body size, we employed genetically generated female worms in the *fem-1* loss-of-function mutant line, which cannot self-produce progeny because the worms are devoid of sperm.^28^ We crossed these worms with wild-type (WT) males of different ages (Fig. 1h, i). In this sex-specific parental age analysis, we observed significant changes in offspring adult size upon the alteration of maternal (Fig. 1h) but not paternal age (Fig. 1i), suggesting that the parental age effect on offspring adult size in *C. elegans* is attributable to the MAE.

In the MAE transmission investigation, we found that the MAE on offspring adult size could not be passed to the F2 generation from the F1 animals (Supplementary information, Fig. S5d), suggesting an intergenerational property of the MAE, which is in line with the findings of a previous study.^4^ Intriguingly, we also observed no correlation between MAE and offspring lifespan in *C. elegans* (Supplementary information, Fig. S5e and Table S1), which contrasts with proposals of earlier studies that aged mothers produce short-lived offspring in other organisms.^1,29^ Furthermore, we noticed that the offspring of aged mothers had significantly lower fat deposits and better chemotactic responses (Supplementary information, Fig. S5f, g) but behaved less consistently when confronting temperature challenge than those of young mothers (Supplementary information, Fig. S5h). Altogether, consistent with the observations in humans and flies, these results demonstrate a clear and robust correlation between maternal age and offspring adult traits in *C. elegans*.

### MAE delivery via passage of donut-shaped mitochondria

It has been reported that a vitellogenin-2 (VIT-2)-mediated yolk-provisioning mechanism accounts for MAE-mediated size regulation in *C. elegans* larvae.^23^ To explore whether the MAE on the adult size of offspring also depends on VIT, we conducted MAE experiments in multiple lines of animals with a single mutation in the *vit-2* and *vit-5* genes or with double or triple mutations in *vit-1*, *vit-2*, *vit-5*, and *vit-6*. Strikingly, MAE appeared to be uninterruptedly transmitted into offspring to shape their adult sizes in all tested VIT loss-of-function mutant strains (Supplementary information, Fig. S6a–c), indicating an independence of the previously reported yolk-provisioning mechanism for MAE-mediated size changes. Lysosomal function changes with aging in the germline of *C. elegans* and plays a critical role in the avoidance of damage transmission from parents to progeny.^30,31^ It was also recently discovered that parents’ memories formed upon neuronal mitochondrial stress can be passed down to offspring for multiple generations via a novel mitochondrial protein response (UPR^mt^) mechanism.^32^ However, significant size differences were still observed between the OD2 and OD5 offspring of animals bearing the *vps-18* or *atfs-1* mutants (Supplementary information, Fig. S6d, e), which disrupt lysosomal^33^ and UPR^mt^^32^ function, respectively, implicating a novel MAE-mediated mechanism of offspring adult size regulation.

**Fig. 2:**
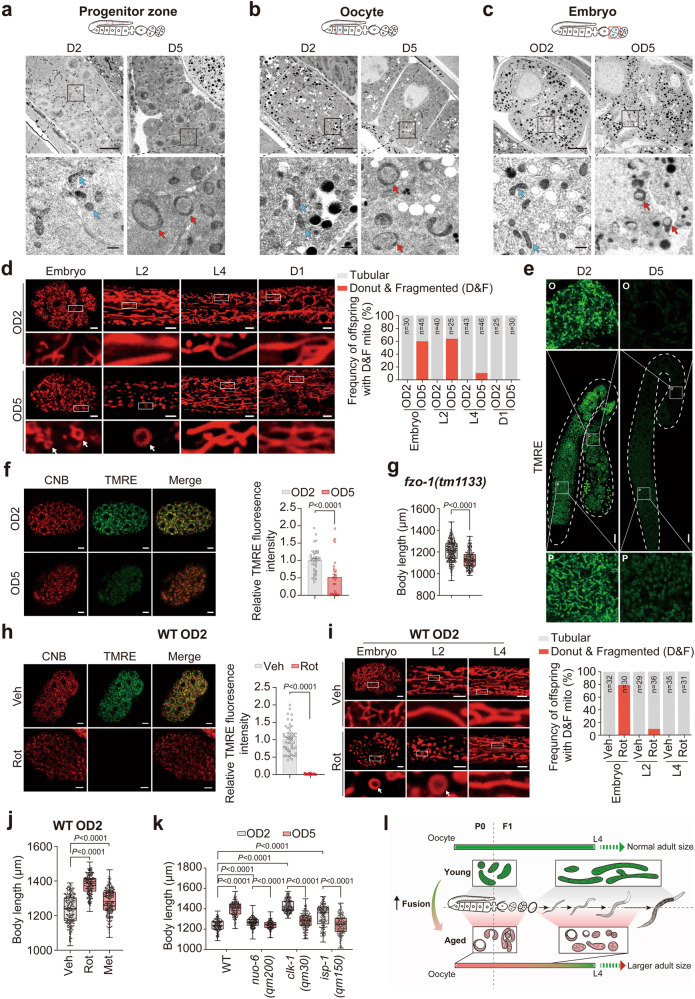
MAE programs the adult body size of offspring through the passage of aged mitochondria. **a**–**c** Representative TEM images of mitochondria (blue arrows, normal; red arrows, donut-shaped) obtained from progenitor zones (**a**), oocytes (**b**) and embryos (**c**) of D2 and D5 worms. The scale bars in the upper panel represent 5 μm, and the lower bars represent 0.5 μm. **d** Confocal images (left) and the quantified ratios (right) of mitochondria stained by cationic Nile blue (CNB, a self-developed fluorescent probe) in OD2 and OD5 animals in the embryonic, larval 2 (L2) or L4 stage. The scale bars represent 5 μm. White arrows indicate donut-shaped mitochondria. The percentage of worms with abnormal mitochondria is shown in the right bar plot. **e** Confocal images of mitochondria in the germline of D2 and D5 worms stained by TMRE. The zoomed-in images were displayed to show clear mitochondrial morphology in the progenitor zones (P) and oocytes (O). Scale bars, 20 μm. **f** Confocal images (left) and the quantified TMRE fluorescence intensities (right) of mitochondria stained by CNB and TMRE in OD2 and OD5 embryos. The scale bars represent 5 μm. **g** Adult body length comparisons between OD2 and OD5 animals of the mitochondrial fusion gene *fzo-1* mutant. **h** Confocal images (left) and the quantified TMRE fluorescence intensities (right) of mitochondria stained by CNB and TMRE in the embryos of rotenone (Rot, 5 μM)-treated D2 WT mothers. ddH_2_O served as the vehicle control (Veh). The scale bars represent 5 μm. **i** Confocal images (left) and the quantified ratios (right) of mitochondria stained by CNB in offspring born to Rot (5 μM)-treated D2 WT mothers. ddH_2_O served as the vehicle control (Veh). White arrows indicate donut-shape mitochondria. The scale bars represent 5 μm. The percentage of worms with abnormal mitochondria, including donut-shaped and fragmented mitochondria, is shown in the right bar plot. **j** Adult body length measurements in offspring from D2 WT mothers treated with 5 μM Rot or 50 mM metformin (Met) for 12 h. **k** MAE-mediated adult body size changes in the offspring of WT and mitochondrial *nuo-6(qm200), clk-1(qm30) or isp-1(qm150)* mutant animals. **l** Model of the mitochondrial basis for MAE to program the adult size of offspring. Dots in box plots represent worm numbers. The data are presented as the means ± SEM with bar plots in **f**, **h** or box plots in **g**, **j**, **k**. In the box plots, the centerline is the median, the box range shows the 25th–75th percentiles, and the whiskers indicate the minimum–maximum values. The box plots (**g**, **j**, **k**) and bar plots (**f**, **g**) were analyzed by unpaired *t*-test. Biological replicates: 3 (**g**, **j**, **k**).

Mitochondria are usually transmitted from mothers to offspring via oocytes. Mitochondrial dysfunction occurs with organismal aging, and aged mothers can pass defective mitochondria into oocytes.^12,13^ It was thus hypothesized that the MAE on the adult size of *C. elegans* may originate from aged mitochondria. We next monitored and compared the mitochondrial inheritance process from the oocyte to embryo stages of *C. elegans* via transmission electron microscopy (TEM) and confocal imaging of oocyte mitochondria in D2 and D5 mothers. We observed that old worms passed a range of abnormally shaped aged mitochondria, especially donut-shaped mitochondria, to their offspring via oocytes (Fig. 2a–c; Supplementary information, Fig. S7a–c). Notably, in the offspring of aged mothers, the donut-shaped mitochondria were completely restored until the adulthood stage of *C. elegans* (Fig. 2d). However, compared with the slow rejuvenation of mitochondrial morphology, the offspring born to aged mothers seemed to exhibit much faster recovery of their mitochondrial DNA (mtDNA) mass and ATP production activity, apparently during the hatching process (Supplementary information, Fig. S8a, b). The rejuvenated mitochondria in OD5 animals appeared to have better metabolic activities, as indicated by their significantly higher oxygen consumption at the basal level (Supplementary information, Fig. S8c) and slightly yet non-significantly higher ATP production (Supplementary information, Fig. S8b) than those of OD2 animals.

Donut-shaped mitochondria have been observed in various conditions, such as axonal boutons of aged monkeys^34^ and cultured cells exposed to mitochondrial stressors,^35,36^ and are considered a peculiar morphological feature of mitochondrial dysfunction.^36,37^ Consistently, our tetramethylrhodamine, ethyl ester (TMRE) staining results indicated that the mitochondria in germlines of aged D5 animals or OD5 embryos had much lower membrane potentials than those in D2 germlines or OD2 embryos (Supplementary information, Fig. 2e, f), suggesting that the aged mitochondria transmitted from old mothers to offspring were dysfunctional with low membrane potentials. The fate or dynamics of donut-shaped mitochondria have been characterized in cultured cells: donut-shaped mitochondria are formed in response to mitochondrial stress through autofusion or interfusion mechanisms but are restored upon removal of mitochondrial stressors in a fusion-dependent manner.^36^ It remains unclear whether mitophagy is involved in the dynamics of donut-shaped mitochondria. In this study under MAE conditions, we did not observe donut-shaped mitochondria present in either OD2 or OD5 embryos of the *fzo-*1 mutants that are defective in mitochondrial fusion (Supplementary information, Fig. S9a). Strikingly, those donut-shaped mitochondria markedly accumulated in all tested stages of both OD2 and OD5 animals of the *drp-1* or *pink-1* mutants with defects in mitochondrial fission or mitophagy (Supplementary information, Fig. S9b, c). Moreover, in the body size comparisons, we found that the MAE was successfully transmitted in mitochondrial fission and mitophagy mutants but not in the mitochondrial fusion mutant animals, which cannot produce and pass donut-shaped mitochondria down to their offspring (Fig. 2g; Supplementary information, Fig. S9d, e). This strongly indicates the indispensable role of donut-shaped mitochondria in the MAE delivery.

It has been reported that donut-shaped mitochondria can be generated via inhibition of mitochondrial energetics by mitochondrial electron transport chain (ETC) inhibitors in cultured cells.^36,37^ We also observed that transient treatment of D1 mothers with the complex I inhibitor rotenone resulted in the aggregation of donut-shaped mitochondria with reduced membrane potential in OD2 offspring (Fig. 2h), which showed a gradual recovery in mitochondrial morphology similar to that of OD5 animals along the progression of their developmental stages (Fig. 2i). Thus, it is possible that inhibition of mitochondrial complex I by rotenone or metformin^38^ in D2 mothers may phenocopy the MAE on offspring adult size. Indeed, we found that the adult lengths of offspring were significantly increased by supplementation of both drugs to mothers of a young age (Fig. 2j). Furthermore, it was noted that mitochondrial dysfunction produced via mutation of genes encoding the demethoxyubiquinone monooxygenase that is required for the biosynthesis of coenzyme Q (*clk-1)*, the subunit of mitochondrial complex I (*nuo-6*) and the iron sulfur protein of mitochondrial complex III (*isp-1*)^39^ also resulted in evident aggregation of donut-shaped mitochondria and similar mitochondrial rejuvenation processes in both OD2 and OD5 of those mutants (Supplementary information, Fig. S10a–c). Notably, we found that these mutant OD2 offspring animals had significantly larger adult body sizes, although their body sizes were smaller during the developmental stages than those of WT worms (Fig. 2k; Supplementary information, Fig. S11), possibly due to their mitochondrial deficiencies preventing the production of sufficient energy for early-life growth demand. Remarkably, mutations of *nuo-6*, *clk-1*, or *isp-1* not only inhibited the MAE-induced size increase but also resulted in reduced body length of the offspring born to aged mutant mothers (Fig. 2k). Unfortunately, the mechanism by which MAE triggered the reduction in the body length of offspring produced by aged mitochondrial mutants remains unidentified. Collectively, these results suggest that the mitochondria inherited from aging mothers and their rejuvenation in developing offspring have a lifelong impact on the size of the adult offspring (Fig. 2l).

### AMPK as an MAE sensing-relaying switch

**Fig. 3:**
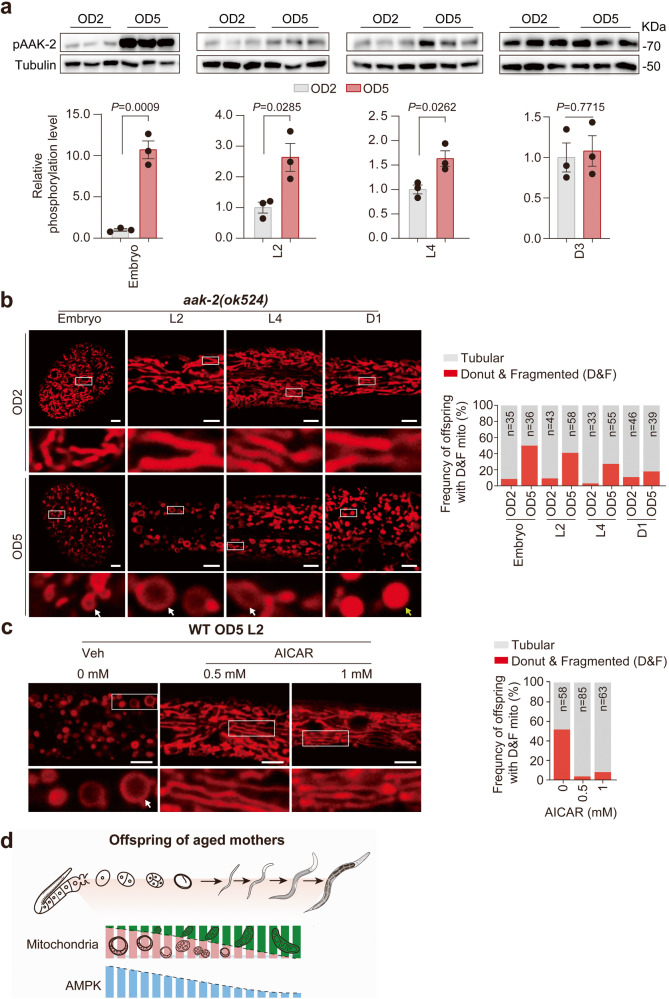
Activation of AMPK in the early life of offspring drives the rejuvenation of inherited aberrant mitochondria. **a** Western blot analysis of AAK-2 phosphorylation levels in OD2 animals and OD5 animals in the embryonic, larval 2 (L2), L4 and adult stages. Representative immunoblots are displayed in the upper panel, and the quantification results are shown with bar plots in the lower panel. Dots in the bar plot represent biological replicates. **b** Confocal images (left) and the quantified ratios (right) of mitochondria stained by CNB in *aak-2(ok524)* mutant offspring (OD2 and OD5) in the embryonic, L2 or L4 stage. The scale bars represent 5 μm. White arrows indicate donut-shaped mitochondria, and yellow arrow indicate fragmented mitochondria. **c** Quantified ratios of mitochondria stained by CNB in WT OD5 animals in the L2 stage. The scale bar represents 5 μm. The percentage of worms with abnormal mitochondria is shown in the right bar plot. **d** Model of the mitochondria-AMPK interplay mediating the shaping of offspring adult size by the MAE. The data are presented as the means ± SEM with bar plots in **a**. The bar plots (**a**) were analyzed by unpaired *t*-test. Biological replicates: 3 (**a**).

AMPK is a well-established sensor and regulator of mitochondrial activity and dynamics;^40^ thus, we next monitored whether offspring AMPK activity changes in response to maternal aging. Surprisingly, we found that the AMPK activity of offspring born to aged mothers was higher in early life than that of offspring born to young mothers, particularly during embryogenesis where the MAE activated AMPK without increasing the total AMPK protein level (Supplementary information, Fig. S12a), but not in the D3 adult stage (Fig. 3a). This stage pattern seems to correlate with the mitochondrial rejuvenation process (Fig. 2d). We then explored and confirmed the indispensable role of AMPK in mitochondrial rejuvenation in offspring from aged mothers. The mitochondrial recovery process was markedly delayed when AMPK was mutated (Fig. 3b) but was greatly promoted upon AMPK activation by 5-aminoimidazole-4-carboxamide riboside (AICAR) treatment (Fig. 3c; Supplementary information, Fig. S12b). Altogether, these results suggest that hyperactivation of AMPK in offspring is attributable to aberrant mitochondria inherited from aged mothers, while in turn, activated AMPK is responsible for the rejuvenation of aberrant mitochondria (Fig. 3d).

**Fig. 4:**
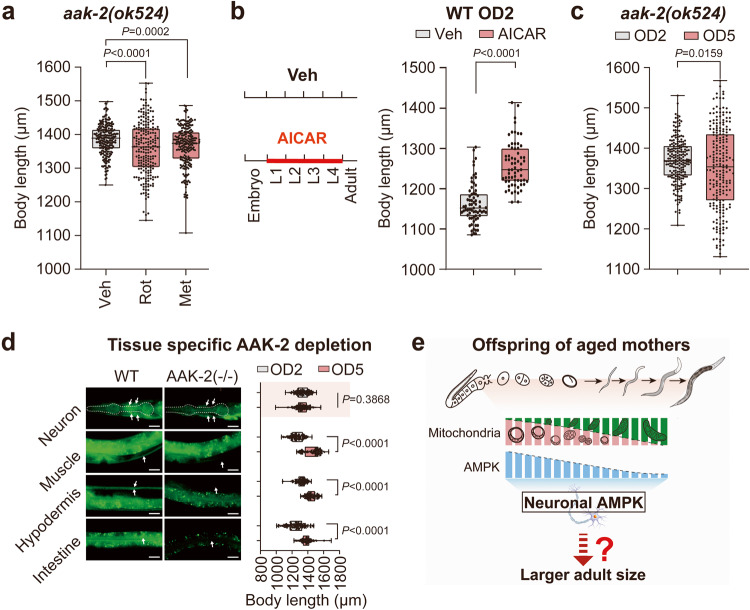
Activation of neuronal AMPK in early life is required for the MAE to program offspring body size in adulthood. **a** Adult body length measurements in offspring born to D2 *aak-2(ok524)* mutant worms treated with 5 μM rotenone (Rot) or 50 mM metformin (Met) for 12 h. ddH_2_O served as the vehicle control (Veh). **b** Adult body length changes in WT animals born to D2 mothers in response to early-life treatment from the larval 1 (L1) stage to the L4 stage with 1 mM AICAR. **c** Body length comparison between OD2 and OD5 *aak-2(ok524)* mutant animals. **d** Tissue-specific role of AMPK in mediating the MAE on offspring body size. The experiment was performed with ZIF-1-mediated protein degradation of GFP-fused AAK-2. The success of tissue-specific depletion of AMPK was indicated by loss of GFP fluorescence in the indicated tissues (white arrows) in the left images. The adult body lengths of OD2 and OD5 animals with the indicated tissue-specific AMPK depletion are compared as listed in the right panel. The scale bars represent 20 μm. **e** Model of the mitochondria-AMPK interplay in mediating the shaping of offspring adult size by MAE. Dots in box plots represent worm numbers. Box plots in **a**–**d**: the centerline is the median, the box range shows the 25th–75th percentiles, and the whiskers indicate the minimum–maximum values. The numbers (*n*) of total tested animals for each group are shown beneath the *x*-axis. The box plots (**a**–**d**) were analyzed by unpaired *t*-test. Biological replicates: 3 (**a**–**d**).

As both rotenone and metformin can indirectly stimulate AMPK activation by inhibiting complex I,^41^ we hypothesized that these two drugs may induce an adult size increase in *C. elegans* by activating AMPK signaling. Indeed, both drugs displayed a size-increasing effect in WT OD2 animals (Fig. 2j); however, such effect was ablated in the *aak-2(ok524)* AMPK loss-of-function OD2 mutants (Fig. 4a). This observation demonstrated full AMPK activity dependency of the size-increasing effect induced by mitochondrial complex I deficiency. Remarkably, we detected that AMPK was significantly activated in offspring born to rotenone-treated WT animals and all tested mitochondrial mutant OD2 offspring (Supplementary information, Fig. S12c, d), while the MAE did not further increase the phosphorylation of AMPK in OD5 offspring of either the *clk-1* or *nuo-6* mutant animals (Supplementary information, Fig. S12e, f). Meanwhile, direct activation of AMPK by treatment with AICAR in the early-life stages of the offspring born to young WT mothers also significantly increased their adult sizes (Fig. 4b). Furthermore, AMPK deficiency via mutation of *aak-2* completely abolished the MAE-mediated offspring adult size increase and even yielded OD5 animals that were smaller than OD2 animals (Fig. 4c). To further identify the specific tissues in which AAK-2 activity transmits the MAE for adult size regulation in offspring, we depleted AAK-2 in various tissues of *C. elegans*, including neurons, muscle, hypodermis, and intestine. Strikingly, we found that only neuronal AMPK was required for MAE-mediated offspring adult size regulation (Fig. 4d), indicating the existence of a signal downstream of neuronal AMPK responsible for MAE-mediated size regulation (Fig. 4e).

### MAE implementation requires TGFβ signaling

To explore the downstream elements of AMPK that account for MAE-mediated body size changes, we first investigated the two classical pathways regulated by AMPK, the insulin/insulin-like growth factor (IGF) and target of rapamycin complex 1 (TORC1) signaling pathways.^42^ Using *daf-2*, *daf-16* and *rsks-1* mutant animals, which have compromised IGF or TORC1 signaling activity,^42,43^ we ruled out the dependence of these two pathways for MAE-mediated size regulation (Supplementary information, Fig. S13a–c).

**Fig. 5:**
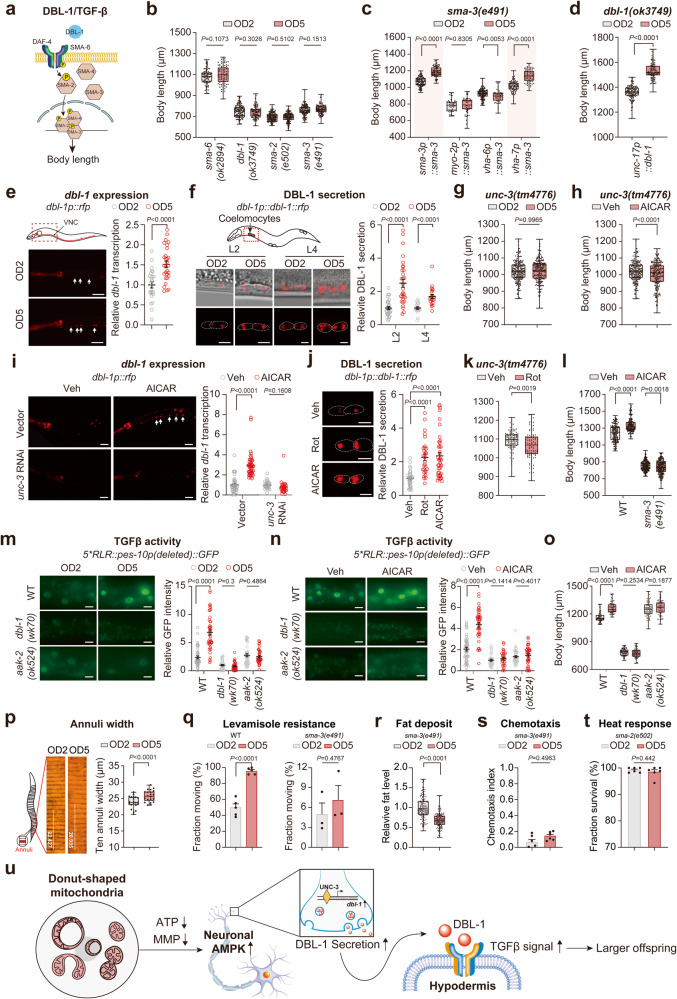
The mitochondria-AMPK-DBL-1/TGFβ axis is indispensable for MAE-mediated offspring adult size shaping. **a** Schematic illustration of *C. elegans* DBL-1/TGFβ pathway. **b** Adult body length comparisons between OD2 and OD5 animals from the indicated DBL-1/TGFβ signaling mutant mothers. **c** Adult body length comparisons between OD2 and OD5 animals born to *sma-3(e491)* mutant mothers with the indicated global (*sma-3*) or tissue-specific SMA-3 rescue in the pharynx (*myo-2*), intestine (*vha-6*) and hypodermis (*vha-7*). **d** Adult body length comparisons between OD2 and OD5 offspring of *dbl-1(ok3749)* mutant mothers with a neuron-specific rescue driven by the *unc-17* promoter. **e**, **f** The expression of *dbl-1* gene (**e**) and the secretion of DBL-1 protein (**f**) in OD2 and OD5 animals in the indicated stages. The scale bars represent 20 μm (**e**) and 5 μm (**f**), respectively. **g**, **h** Adult body length measurements in *unc-3(tm4776)* mutant animals challenged by MAE (**g**) or treated with AICAR (**h**) in early-life stages (L1–L4). **i** The transcriptional changes of *dbl-1* indicated by the *dbl-1*-RFP reporter upon treatment with indicated RNAi and/or compound from P0 mothers to F1 L2 stage. The scale bars represent 20 μm. **j** Relative DBL-1 secretion in L2 offspring from mothers with the indicated treatments. Rotenone (Rot, 5 μM) or AICAR (1 mM) was applied. The examined head coelomocytes are highlighted with white dotted lines. The scale bars represent 5 μm. **k** Adult offspring body length measurements in *unc-3(tm4776)* mutant animals treated with 5 μM Rot at D2 maternal stage for 12 h. **l** Adult body length measurements in offspring of WT and *sma-3(e491)* mutant mother worms treated with 1 mM AICAR or vehicle (Veh). **m**, **n** DBL-1/TGFβ signaling activity test using the RAD-SMAD reporter in OD2 and OD5 animals (**m**) or AICAR-treated offspring (**n**) born to WT, *dbl-1(wk70)* or *aak-2(ok524)* mutant animals. The scale bars represent 5 μm. **o** Adult body length measurements in OD2 animals of WT, *dbl-1(wk70)* or *aak-2(ok524)* mutant mothers. These OD2 animals were treated with 1 mM AICAR or Veh in early-life stages. **p** Annulus width measurements in tail regions of OD2 and OD5 worms. **q** Levamisole sensitivity assay for OD2 and OD5 animals born to WT or *sma-3(e491)* mutant mothers. **r** Fat deposit assay for OD2 and OD5 animals born to *sma-3(e491)* mutants. **s** Chemotaxis assay for OD2 and OD5 animals born to *sma-3(e491)* mutants. **t** Heat response assay for OD2 and OD5 animals born to *sma-2(e502)* mutants. **u** Model of MAE-mediated offspring adult size shaping. Dots in box plots represent worm numbers, and dots in bar plots indicate biological replicates. The data are presented as the means ± SEM with bar plots (**q**, **s**, **t**), box plots (**b**–**d**, **g**, **h**, **k**, **l**, **o**) or scatter dot plots (**e**, **f**, **i**, **j**, **m**, **n**). In the box plots, the centerline is the median, the box range shows the 25th–75th percentiles, and the whiskers indicate the minimum–maximum values. The bar plots (**q**, **s**, **t**), box plots (**b**–**d**, **g**, **h**, **k**, **l**, **o**) and scatter dot plots (**e**, **f**, **i**, **j**, **m**, **n**) were analyzed by unpaired *t*-test. Biological replicates: 4 (**b**), 3 (**c**–**r**), 5 (**s**, **t**).

TGFβ signaling is also a well-known regulator of organismal size.^44^ There are two TGFβ signaling pathways in *C. elegans* (Fig. 5a; Supplementary information, Fig. S13d). One is the DBL-1/TGFβ signaling pathway, which is mainly responsible for body size regulation; the other is the DAF-7/TGFβ signaling pathway, which is involved in dauer formation, metabolism, and aging regulation.^45^ Remarkably, we found that the MAE-mediated adult offspring size increase solely depended on the DBL-1/TGFβ pathway rather than the DAF-7/TGFβ pathway (Fig. 5b; Supplementary information, Fig. S13e). Furthermore, we demonstrated that the MAE on offspring adult size regulation in *sma-3(e491)* mutant animals could be restored by either the global or hypodermis-specific rescue of SMA-3 activity (Fig. 5c), strongly indicating that hypodermal DBL-1/TGFβ activity is essential for MAE implementation to influence offspring adult size.

DBL-1, a ligand of the DBL-1/TGFβ pathway, is expressed and secreted from neurons and acts in hypodermal cells to regulate growth and development.^46^ We found that expressing DBL-1 driven by a neuron-specific *unc-17* promoter in *dbl-1* mutant animals restored not only the mutant’s body length but also the MAE on body size increase outcomes (Fig. 5d; Supplementary information, Fig. S13f), indicating that the MAE acts through neuronal regulation, likely by modulating DBL-1. To examine whether the expression of *dbl-1* and its secreted protein product are altered by the MAE, we created two *dbl-1* transgenic lines using the native promoter: one with only red fluorescent protein (RFP), which was used to monitor the transcriptional activity of *dbl-1*; and the other one expressing RFP-fused DBL-1 protein, which enabled detection of secreted DBL-1 via quantification of the accumulated RFP patches in coelomocytes of *C. elegans*.^46^ We found that both the expression of *dbl-1* and the levels of the secreted DBL-1 protein in larval offspring were significantly elevated with increasing maternal age (Fig. 5e, f), suggesting that the MAE may modulate the activity of DBL-1 by altering its expression at the mRNA level. It has been documented that the expression of *dbl-1* is controlled by UNC-3, a phylogenetically conserved transcription factor regulating cholinergic motor neuron activities.^47^ Intriguingly, we observed that the MAE or AICAR treatment could not induce body size changes in offspring born to *unc-3* mutant animals (Fig. 5g, h). RNAi knockdown of *unc-3* completely blocked the effect of AICAR in increasing the expression of *dbl-1* (Fig. 5i), indicating that neuronal AMPK regulates *dbl-1* expression in a UNC-3-dependent manner. We also found that activation of TGFβ signaling by RNAi knockdown of the signaling suppressor gene *lon-2*^48^ significantly increased the body size of AMPK mutant OD2 animals and restored the MAE-mediated size increase in the mutant OD5 animals (Supplementary information, Fig. S13g). Furthermore, we observed that DBL-1 secretion was stimulated through administration of either rotenone for inhibition of complex I or AICAR for activation of AMPK (Fig. 5j). However, the boost in offspring body size caused by either rotenone or AICAR in WT animals (Figs. 2j and 4b) was completely abolished by the loss-of-function mutation in *sma-3* (Fig. 5k, l). Together, our findings indicate the existence of active epistatic interactions among mitochondria, AMPK and the DBL-1-directed TGFβ signaling pathway for MAE implementation in offspring.

To investigate whether the activity of DBL-1/TGFβ signaling in offspring is faithfully stimulated by MAE, we employed a previously established in vivo RAD-SMAD reporter line.^46^ Interestingly, we found that MAE or AICAR treatment significantly elevated TGFβ activity in WT offspring but not in *dbl-1* or *aak-2* mutants (Fig. 5m, n), while the deficiency in either *dbl-1* or *aak-2* completely suppressed the effect of AICAR in increasing the adult body sizes of *C. elegans* (Fig. 5o), supporting the idea that an increase in DBL-1 secretion is necessary for MAE-mediated offspring body size regulation. It has been documented that the activity of TGFβ can be assessed by measuring the annulus width and the response to the cholinergic agonist levamisole in worms.^49^ Thus, we conducted these phenotypic experiments and found that the administration of MAE resulted in an increase in annulus width and levamisole resistance in WT but not in *sma-3* mutant animals with TGFβ signaling dysfunction (Fig. 5p, q). These results suggest that TGFβ signaling was activated by the MAE in WT animals but not in those with *sma-3* mutations. Although the decreased fat deposit induced by the MAE (Supplementary information, Fig. S5f) seemed to be independent of TGFβ signaling (Fig. 5r), the MAE-mediated responses to environmental stimuli (Supplementary information, Fig. S5g, h) were completely abolished by mutations in genes downstream of TGFβ signaling (Fig. 5s, t). Together, these results suggest that the mitochondria-AMPK-TGFβ axis is indispensable for the MAE to program traits of adult offspring (Fig. 5u).

## Discussion

Maternal age has been both negatively and positively associated with numerous health outcomes in offspring,^5^ but the underlying mechanisms are not fully understood. In the present study, we found that the MAE is an ancient factor shaping offspring adult size, at least in the three species studied. Mechanistically, we revealed that the MAE is established via mitochondrial inheritance and implemented through the mitochondria-AMPK-TGFβ signaling axis to program the adult body size of offspring. Larger offspring are generally found to have better access to depleted resources across taxa.^50^ Thus, from an evolutionary perspective, the MAE may help offspring better prepare for future challenges.^51^

In the human data analysis, we observed diverse effects of maternal age on multiple offspring adult phenotypes, including positive impacts on cognitive ability and forced vital capacity, and a negative influence on body fat deposit. These findings promoted our understanding of the correlation between maternal age and adult offspring outcomes. Nonetheless, these associations could potentially be influenced by confounding factors. We have considered and adjusted for several essential confounding factors, including age, sex, Townsend deprivation index (TDI), and educational attainment (EA) of the offspring, but unfortunately, the corresponding information was unavailable for the parents. We reason that socioeconomic factors, to some extent, can be captured by population stratification, which can be corrected by fitting a genetic relationship matrix derived from common genetic variants in a linear mixed model (LMM).^52^ The result showed that the LMM estimate of the MAE remained highly significant for height (*β* = 0.035, *P* = 5.88e–29). Importantly, how other confounding factors that may manipulate maternal mitochondria, such as smoking and drinking during pregnancy, would impact the MAE-mediated adult trait regulation remains to be explored in humans.

The impact of maternal aging on mitochondrial dynamics and function has been extensively explored in oocytes of various organisms^13^ but not in embryos or offspring in developmental stages. Consistent with previous studies in mouse, hamster and human oocytes,^12,53^ our work demonstrated that old worms can also pass aged donut-shaped mitochondria with low membrane potentials to their offspring via oocytes, which presumably resulted in lower mtDNA content and lower ATP levels in their offspring embryos than those in offspring embryos born to young worms. We observed, for the first time, that donut-shaped mitochondria can be passed via the germline and oocytes of aged worms into their offspring embryos and play an important role in organismal adult trait development. Neurogenesis, which is a high energy-demanding activity, occurs heavily during the early development of *C. elegans*. Concurrently in the early life of offspring born to aged mothers, it is very likely that the dysregulated function of donut-shaped mitochondria will lead to activation of neuronal AMPK (Fig. 5u).

Our results demonstrating the indispensable role of mitochondrial fusion process in donut-shaped mitochondria formation and the gradual mitochondrial recovery process in developmental stages of live animals align well with the in vitro finding that the donut-shaped mitochondria are formed via mitochondrial autofusion or interfusion and can be restored upon treatment termination.^36^ However, future work is still needed to mechanistically explain (1) how the mitochondria-AMPK interplay occurs in physiological conditions; (2) whether developmental reactive oxygen species play any role in such interplay, since they accompany mitochondrial rejuvenation and have been reported to play physiological roles^54^; and (3) whether the rejuvenation or clearance of donut-shaped mitochondria involves mitophagy, despite that we have demonstrated its dispensable role for MAE delivery and exertion. The molecular machinery underlying how neuronal AMPK increases the expression of *dbl-1* by modulating UNC-3 needs to be explored in the future study. In addition to genetic inheritance, our study proposes the contribution of mitochondrial inheritance to adult body size determination, likely through mitochondrial energetic status beyond mtDNA inheritance. Whether other traits related to the MAE can be totally explained by mitochondrial inheritance warrants further investigation. Whether MAE and the proposed signaling axis are conserved in other species, such as mouse models, and what role the MAE may play in offspring disease susceptibility also remain to be explored. Our study provides a foundation for future research and the development of potential interventions for reprogramming adult health outcomes.

## Materials and methods

### Human data analysis

This study was approved by the Ethics Committee of Westlake University (approval no. 20200722YJ001).

We used human data from the UK Biobank, a large cohort study consisting of ~500,000 participants aged between 40 and 69 at recruitment with extensive phenotypic records.^55^ Each individual who participated in the UK Biobank study provided written informed consent prior to their involvement in the research. In this study, we selected 349,662 unrelated individuals of European ancestry based on relatedness inferred from genome-wide SNP data and excluded the adopted individuals.

To investigate the association between maternal age and offspring adult height, we performed a linear regression analysis, with age (offspring), age^2^, sex, EA, TDI, and the first 10 principal components (PCs) derived from genetic data fitted as covariates. After exclusion of individuals with missing maternal age, 135,054 individuals were included in the model: height ~ MAE + age + age^2^ + sex + EA + TDI + PCs. Moreover, the same analysis was applied to investigate the relationship between paternal age and offspring adult height with 77,553 individuals included in the model: height ~ PAE + age + age^2^ + sex + EA + TDI + PCs. To determine whether the parental age effect on offspring adult height is driven by maternal or paternal age, we fitted maternal and paternal ages jointly in a model with 56,606 individuals: height ~ MAE + PAE + age + age^2^ + sex + EA + TDI + PCs. We also performed a regression analysis by stratifying the parent–offspring pairs by paternal (or maternal) age to investigate the relationship between maternal (or paternal) age and offspring adult height. To account for a potential nonlinear relationship between maternal age and offspring adult height, we assessed the association by a polynomial regression model: height ~ MAE + MAE^2^ + MAE^3^ + age + age^2^ + sex + EA + TDI + PCs. Furthermore, we assessed the associations between maternal age and a host of phenotypes by a linear regression model: phenotype ~ MAE + age + age^2^ + sex + EA + TDI + PCs.

### Offspring fly preparation

There are no ethical concerns associated with the use of flies. All flies were maintained on cornmeal medium containing cornflour, sucrose, brown sugar, agar, yeast, propionic acid, and Nipagin M in an incubator (Forma™ Environmental Chamber Model 3949, Thermo Scientific) with a 12:12 h light/dark cycle at 25 °C.

To reduce mother age difference-induced variations in offspring, newly hatched virgin females with the genotype of interest were crossed with newly hatched males bearing the same genotype in bottles containing the medium mentioned above to synchronize the age and status of offspring in the next generation, and flies were transferred into fresh bottles every 3 days. The newly hatched virgin females, considered parent flies (the P0 generation), were collected and immediately transferred into new bottles, with 100 flies in each bottle. Flies were then transferred into fresh bottles every other day. Virgin females were reared either for 5 days (young, referred to as D5) or 20 days (aged, referred to as D20) and were considered young and old mothers, respectively. Three-day-old male flies with the genotype of interest were collected and crossed with D5 or D20 virgin females for 24 h in small vials, each containing 5 male flies and 6 female flies. *w*^1118^ virgin males were reared either for 5 days (young, referred to as D5) or 20 days (aged, referred to as D20) and considered young or old fathers, respectively. D5 *w*^1118^ virgin female flies were collected and crossed with D5 or D20 virgin males for 24 h in small vials, each containing 5 male flies and 6 female flies. The parental flies (P0) were allowed to lay eggs for 24 h. After eclosion, the F1 generation flies were raised on normal food for 3 days, and then males and females were separated and stored in 2-mL tubes at –80 °C for cryopreservation. Each experiment was repeated three times independently.

To test whether mating times influence MAE exertion, *w*^1118^ virgin female flies from the identical fertilized batch were collected and divided into two groups: 1) young, virgin female flies were cultured alone for 5 days, referred to as D5 (V); 2) aged, virgin female flies were cultured alone for 20 days, referred to as D20 (V). On Day 5, virgin flies from the D5 group were mated with young male flies. On Day 20, the non-virgin female flies from the D5 group were mated again with young male flies, referred to as D20 (NV), and the virgin flies from D20 (V) group were also mated with young male flies. Each vial contains 6 female flies and 5 male flies (3-day-old), and females were allowed to lay eggs for 24 h in all groups. Then all the male flies were removed and discarded. After eclosion, the offspring flies were raised on normal food for 3 days, and then males and females were separated and stored in 2-mL tubes at –80 °C for cryopreservation. Each experiment was repeated three times independently.

### Size and body mass measurements and imaging in flies

The wings of adults with the genotype of interest were carefully dissected, placed in a droplet of Canada balsam (Sigma) on a microscope slide and covered with a coverslip. The wings were imaged using a stereomicroscope (Olympus), and the size was measured with Adobe Photoshop CC 2019 (version 20.0.5). For head and body measurements, 3-day-old flies were frozen at –80 °C and tapped vigorously in a 2-mL tube to separate the body and head; the legs and wings were discarded. The adult heads and bodies were fixed on white paper with double-sided tape and imaged with a stereomicroscope (Olympus). The head size, thorax length, abdomen length and body (thorax + abdomen) length were measured using the life science imaging software package Olympus cellSens Dimension (version 2.3). Adult fly body mass was measured using electronic analytical balance (Techcomp, XJ220A).

### *C. elegans* strains and culture

The WT Bristol N2, *fem-1(hc17) IV*, *dbl-1(ok3749) V*, *sma-2(e502) III*, *sma-3(e491) III*, *sma-6(ok2894) II*, *daf-8(e1393) I*, *daf-2(e1370) III*, *daf-16(mu86) I*, *rsks-1(ok1255) III*, *vit-2(ok3211) X*, *vit-5(ok3239) X*, *aak-2(ok524) X*, *nuo-6(qm200) I, clk-1(qm30) III, isp-1(qm150) IV, drp-1(tm1108) IV, fzo-1(tm1133) II, pink-1(ok3538) II, atfs-1(gk3094) V, vit-1(hq532);vit-2(ok3211) X, vps-18(tm1125) II, unc-3(tm4776) X*, CS628 *sma-3(wk30) III;qcIs55 [vha-7p::gfp::sma-3 + rol-6(su1006)]* and LW2308 *dbl-1(wk70) V;jjIs2277 [pCXT51(5X RLR::pes-10p(deleted)::gfp) + LiuFD61(mec-7p::rfp)]* strains were used, most of which were obtained from the *Caenorhabditis* Genetics Center. Integrated lines of WLU69 *alyIs1[dbl-1p::rfp + myo-2p::gfp]* and WLU73 *alyIs2[dbl-1p::dbl-1::rfp + myo-2p::gfp]* were generated in this study. The DBL-1/TGFβ reporter strain WLU67 was generated by crossing LW2308 *dbl-1(wk70) V;jjIs2277* with N2. WLU233 *alyTi1[unc-17p::dbl-1::dbl-1 3*′*utr + cb unc-119(+) + ttTi5605 + NeoR(+) + unc-18(* + *)] I;unc-119(ed3) III;dbl-1(ok3749) V* was created in this study. GSW308 *aak-2[zac308(aak-2::gfp)]* was a gift from Dr. Wei Zou. DCL979 *mkcSi61 [rgef-1p::vhhGFP4::zif-1CDS::mCherry::his-11::tbb-2 3*′*UTR + Cbr-unc-119(* + *)] II;aak-2 [mkc12(aak-2::gfp::3×flag)] X*, DCL980 *mkcSi62 [myo-3p::vhhGFP4::zif-1CDS::mCherry::his-11::tbb-2 3*′*UTR + Cbr-unc-119(* + *)] II;aak-2 [mkc12(aak-2::gfp::3×flag)] X*, DCL981 *mkcSi63 [dpy-7p::vhhGFP4::zif-1CDS::mCherry::his-11::tbb-2 3*′*UTR + Cbr-unc-119(* + *)] II;aak-2 [mkc12(aak-2::gfp::3×flag)] X*, DCL983 *mkcSi65 [elt-2p::vhhGFP4::zif-1CDS::mCherry::his-11::tbb-2 3*′*UTR + Cbr-unc-119(* + *)] II;aak-2 [mkc12(aak-2::gfp::3×flag)] X* and DCL242 *aak-2 [mkc12(aak-2::gfp::3×flag)] X* were constructed by the Di Chen lab. The strains driven by the *sma-3* native promoter *sma-3(wk30) III;him-5(e1490) V;qcEx24 [sma-3p::gfp::sma-3* + *rol-6(su1006)]*, the pharyngeal-specific *sma-3(wk30) III;him-5(e1490) V;qcEx52 [myo-2p::GFP::sma-3* + *rol-6(su1006)]*, and the intestinal-specific *sma-3(wk30) III;him-5(e1490) V;qcEx53 [vha-6p::GFP::sma-3* + *rol-6(su1006)]* were gifts from Yong Yu. All worms were cultured at 20 °C on nematode growth medium (NGM) plates (containing 5 g/L peptone) seeded with *Escherichia coli* OP50, except that *fem-1(hc17)* mutants were cultured at 25 °C from embryo to young adult to prevent spermatogenesis.

### Construction of the strain with mutation of multiple *vit* genes

The *vit-2(ok3211);vit-5(aly1);vit-6(aly2)* triple mutant line was established through CRISPR/Cas9 genome editing. Briefly, the *vit-5* and *vit-6* genes were edited in the *vit-2(ok3211)* line by coinjecting a mixture of *vit-5* sgRNA (5′-TCTCTTCCGAAGATGCCGAG-3′), *vit-6* sgRNA (5′-TTCAATGGCCAGCTCTCTGC-3′) and CRISPR/Cas9 ribonucleoprotein complex. *rol-6* (pRF4) served as a co-injection marker for identifying target mutants after random genomic repair.

### Offspring preparation in *C. elegans*

Nine-centimeter NGM plates (5 g/L peptone) were prepared and dried after being seeded with 2 mL of bacterial solution for one week at room temperature, which was sufficient to maintain ~5000 synchronized L1s until Day 1 of adulthood (~12–16 h post late L4 stage). To produce OD5 offspring, ~5000 synchronized P0 L1s per plate were prepared. P0 D1 animals were washed off from the plates with M9T buffer (M9 buffer supplemented with 0.05‰ Triton X-100) before starving and were then transferred to new plates. During the transfer, worms with M9T buffer were collected into a 15-mL tube and pelleted by gravity. The eggs and L1 larvae in the supernatant were removed. The worm pellets were resuspended in M9T buffer and pelleted by gravity again. This process was repeated at least three times to remove nearly all the eggs and L1 larvae. Transfer following the above procedures was conducted every 12 h to remove F1s and keep the P0 generation well-fed. Offspring were generated by egg-laying methods for 2 h on a new plate; specifically, OD2 offspring were collected at 40 h post L4 stage, and OD5 offspring were collected at 112 h post L4 stage. The phenotypes of the F1 offspring were evaluated on Day 3 of adulthood.

### CRISPR/Cas9 allele generation

CRISPR-induced genome engineering with a self-excising drug selection cassette (SEC) was performed to knock-in the GFP fluorescent protein to the C-terminus of AAK-2.^56^ A DNA mix containing the repair template (*aak-2::gfp^sec^3×flag*) (10 ng/µL), two Cas9-sgRNA plasmids (50 ng/µL) and a pharyngeal selection marker, pCFJ90 (*Pmyo-2::mCherry::unc-54-3*′*-UTR*) (2.5 ng/µL) (2.5 ng/µL, Addgene, #19327), was injected into N2 young adults. The ccdB sequences from the FP-SEC vector pDD282 (Addgene, #66823) were replaced by two homologous arms (500–700 bp) to generate the repair template FP-SEC plasmid. The sgRNAs were designed using the CRISPR DESIGN tool (http://crispr.mit.edu) and inserted into the vector pDD162 (Addgene, #47549) with the site-directed mutagenesis kit (TOYOBO, SMK-101). Successful knock-in events were selected by PCR genotyping from hygromycin B (350 µg/mL)-resistant rollers that did not carry the co-injection marker. The SEC was removed by heat shock, and homozygous knock-in alleles were verified by Sanger sequencing of PCR products.

sgRNA 1 sequence: 5′-CTCAAAGAATTGCATAGTCT-3′,

sgRNA 2 sequence: 5′-ACAGAGCATTGTTGGCATCA-3′.

### MosSCI single-copy transgene generation

The tissue-specific *vhhGFP4::zif-1* CDS transgenic strains were constructed using the MosSCI method.^57^ Various tissue-specific promoters and the 3918-bp *vhhGFP4::zif-1* CDS::*mCherry*::*his-11*::*tbb-2*-3′-UTR fragment from the pOD2044-intDEG plasmid (Addgene, #89366)^58^ were PCR-amplified and inserted into the MosSCI vector pCFJ151 with the Pro Ligation-Free Cloning Kit (ABM). The injection mixture contained pCFJ601 (*Peft-3::Mos1* Transposase) (50 ng/µL, Addgene, #34874), the selection marker pCFJ90 (*Pmyo-2::mCherry::unc-54*-3′-UTR) (2.5 ng/µL) and the targeting plasmids (pan-neuronal *Prgef-1::vhhGFP4::zif-1 CDS::mCherry::his-11::tbb-2*-3′-UTR; muscular *Pmyo-3::vhhGFP4::zif-1 CDS::mCherry::his-11::tbb-2*-3′-UTR; epidermal *Pdpy-7::vhhGFP4::zif-1 CDS::mCherry::his-11::tbb-2*-3′-UTR; intestinal *Pelt-2::vhhGFP4::zif-1 CDS::mCherry::his-11::tbb-2*-3′-UTR) (50 ng/µL). EG4322 (*ttTi5605;unc-119*(*ed9*)) worms were injected and then maintained at 25 °C until starved. Free-moving worms without the selection marker were picked as successful insertions and confirmed by genotyping and Sanger sequencing.

The ventral nerve cord (VNC) neuron-specific expression of DBL-1 was driven by the *unc-17* promoter. Briefly, the *dbl-1::dbl-1* 3′UTR driven by the *unc-17* promoter (3696 bp) was subcloned into the pCFJ350 plasmid (Addgene, #34866), which allows targeting of well-defined locations in the *C. elegans* genome by the widely used MosSCI method.^59^ Then, neuronal DBL-1 expression plasmid (20 ng/µL), pCFJ601 (Addgene, #34874, 50 ng/µL), pMA122 (Addgene, #34873, 10 ng/µL), pGH8 (Addgene, #19359, 10 ng/µL), pCFJ90 (Addgene, #19327, 2.5 ng/µL), and pCFJ104 (Addgene, #19328, 5 ng/µL) were mixed and injected into the WLU232 *[oxTi185 I;unc-119(ed3) III, dbl-1(ok3749) V]* strain. Homozygous animals were obtained by following a previously reported standardized protocol.^59^

### Adult body length measurement in *C. elegans*

The maximal adult body lengths of the worms were measured on Day 3 of adulthood as previously described.^27^ Briefly, offspring were washed off the NGM plate and paralyzed on a 2% agar plate (containing 50 mM sodium azide). Approximately 60–70 worms per group were placed straight with a glass pick, and their body lengths were determined using a Nikon stereoscope. The varied sample sizes for the *C. elegans* work of this study were decided based on the ease of sample collection and the availability of related resources, and not predetermined by any statistical method. The data were analyzed using GraphPad Prism 8.0.

### Lifespan assay

Lifespan analysis was conducted following our previous protocol.^38^ Briefly, worms were cultured on NGM plates and seeded with *E. coli* OP50. Fluorodeoxyuridine (FUDR, 50 μM) was used to prevent the generation of F2 animals. Worms were transferred to new FUDR plates on Day 3 and Day 6 by washing. The survival rates were scored every day, with the first day of adulthood set as Day 1. A worm was considered dead when it did not respond to touch. The survival plot and statistical analysis were completed with online OASIS2 resources.^60^

### Fat level analysis

The fixed Nile red (BBI Life Science, 7385-67-3) staining was employed as described previously.^61^ Synchronized 3-day-old adult offspring were collected with M9T buffer and washed three times. The worm pellets were fixed in 40% isopropanol and frozen at –80 °C for more than 2 days. The samples were thawed at room temperature and incubated with 3 µg/mL Nile red for 2 h with shaking in the dark. After staining, the staining solution was replaced with M9T buffer and washed for 30 min with shaking. The relative fat levels of tested worms were obtained from the value of fluorescence intensity for each worm divided by the average fluorescence intensity of all OD2 worms measured in three biological replicates.

### Heat stress assay

Approximately 50 synchronized 3-day-old adults per plate were incubated in a 35 °C incubator for 6 h. Worm survival was evaluated by touching after a 6-h recovery at 20 °C. The average survival ratios and standard error of the mean (SEM) were calculated and analyzed by unpaired *t*-test using GraphPad Prism 8.0.

### Levamisole sensitivity test

Worms were washed with M9T buffer three times before the assay. Approximately 40 synchronized 3-day-old adult offspring were transferred to an NGM plate containing 0.5 mM levamisole. The number of animals with movement, namely, a response to prodding, at 1 h after treatment was recorded. Three independent biological trials were conducted. The percentages of moving and non-moving animals, SEM and *P* values (using the unpaired *t*-test) were determined by GraphPad Prism 8.0.

### TEM sample preparation and imaging

The mitochondrial morphology of the hermaphroditic germline, oocyte and embryo was analyzed with TEM. Samples were prepared following a high-pressure freezing standard protocol. Briefly, worms were picked into 200 μL M9 buffer with 20% BSA and frozen with a high-pressure freezing machine (Leica EM ICE). Then, the samples were freeze-substituted in a Leica EM AFS2 freeze-substitution unit with medium (containing 1% osmium tetroxide, 0.1% uranyl acetate, 10% methanol in acetone) using a program as previously described.^62^ At the end of freeze-substitution, the temperature was increased to 20 °C. The samples were washed in pure acetone 3 times before being infiltrated with increasing concentrations of Epon resin (diluted with acetone): 25% Epon resin for 30 min, 33% for 210 min, 50% for 12 h, 75% for 240 min, and pure resin four times in a 24-h period. The samples were embedded in a mold and cured for 2 days at 60 °C.

For TEM imaging, samples were cut into 70-nm-thick sections using a Leica EM UC7 ultramicrotome and collected on copper grids. The sections were counterstained with 2% uranyl acetate and Sato’s triple lead. All imaging was carried out on a Thermo Fisher Scientific Talos L120C TEM at 80 kV equipped with a 2k × 2k Ceta CCD camera. The percentages of donut-shaped mitochondria and non-donut-shaped mitochondria were quantified and analyzed in a blind manner.

### Mitochondrial staining

*C. elegans* were cultured overnight in the dark at 20 °C on NGM plates containing 2 μM cationic Nile blue (CNB) before producing OD2 or OD5 offspring. The oocytes were collected by dissecting the mother worms in M9 buffer, and embryos were obtained by bleaching the overnight-stained worms. CNB-stained L2 and L4 worms were generated by egg laying for 1 h on NGM plates containing 2 μM CNB in the dark at 20 °C. L2 (28 h post egg laying) and L4 (52 h post egg laying) animals were collected for mitochondrial morphology analysis.

TMRE (Thermo Fisher, T669), a mitochondrial membrane potential sensor, was used to evaluate mitochondrial membrane potential in this study. Mothers treated with 2 μM TMRE were used to compare the mitochondrial membrane potential of the germline. Mothers and offspring treated with 5 μM TMRE were used to compare membrane potential between OD2 and OD5. Fluorescence images were taken using an Axiocam 702 mono camera on a Zeiss LSM800 (Airyscan) confocal microscope with a 63× oil objective.

### Feeding RNAi in *C. elegans*

The RNAi clone against *unc-3* used in this study was isolated from a genome-wide *E. coli* feeding RNAi library and fed to *C. elegans* as described previously.^63^

### Compound treatment

For the mitochondrial morphology assay, WT D1 mothers were treated with 5 μM rotenone (TargetMol, T-2970) and 2 μM CNB dye for 12 h. Offspring were collected for imaging at the embryonic, L2 and L4 stages on rotenone and CNB-free plates. The images of hypodermal mitochondria of L2s were taken with an Axiocam 702 mono camera on a Zeiss LSM800 (Airyscan) confocal microscope using a 63× oil objective. For body length measurement, WT D1 mothers were treated with 5 μM rotenone or 50 mM metformin (Sigma, PHR1084) for 12 h, and embryos were produced on rotenone and metformin-free NGM plates. The maximal adult body lengths of tested animals were measured and quantified.

For the mitochondrial recovery assay, WT OD5 animals were treated with 0.5 mM or 1 mM AICAR (Selleck, S1802) mixed with 2 μM CNB dye from embryos to the L2 stage. The images of hypodermal mitochondria of L2s were taken with an Axiocam 702 mono camera on a Zeiss LSM800 (Airyscan) confocal microscope using a 63× oil objective. For the body length assay, WT OD2 animals were treated with 1 mM AICAR from the embryonic to young adult stages (egg laying start) and then transferred to AICAR-free plates. The maximal adult body lengths of tested animals were measured and quantified.

### Annulus staining

Three-day-old adult offspring were washed with M9 buffer three times before being stained with 30 µg/mL DiI (1,1′-dioctadecyl-3,3,3′,3′-tetramethylindocarbocyanine perchlorate, US EVERBRIGHT, D4010) for ~3 h on a stir plate with continuous shaking at 350 rpm at room temperature. Animals were transferred to a new NGM plate after three washes with M9 buffer. Images were taken with an Axiocam 702 mono camera on a Zeiss LSM800 (Airyscan) confocal microscope using a 63× oil objective.

### Oxygen consumption assay

Mitochondrial oxygen consumption of offspring was evaluated by using a Seahorse XFe96 analyzer at 20 °C. Approximately 150 stage-synchronized L2 worms of each offspring in 200 μL of M9 buffer were transferred into each well of a 96-well microplate and 6 wells for each condition. Carbonyl cyanide 4-(trifluoromethoxy) phenylhydrazone (Sigma-Aldrich, C2920) was used to stimulate the respiratory chain to operate at maximum capacity, and sodium azide (Sigma-Aldrich, C2002) was used to estimate nonmitochondrial respiration. Basal respiration was measured for a total of 48 min by 8-min cycles, which included a 3-min mix, a 2-min time delay and a 3-min measurement. Student’s *t*-test was used to calculate the statistical significance.

### ATP level measurement

To quantify the ATP levels of embryos and worms, ~50,000 embryos, 20,000 L2s and 2000 L4s were collected. Worm pellets were boiled for 15 min at 95 °C in a metal bath to release ATP and destroy ATPase activity before three freeze/thaw cycles (from –80 °C to room temperature). The samples were centrifuged at 16,000× *g* for 10 min at 4 °C. The supernatant was carefully transferred to a new precooled tube. All samples were diluted ten times with double-distilled water and then subjected to ATP measurement using an ATP detection kit (Thermo Fisher, A22066) according to the manufacturer’s instructions. The ATP levels were normalized to total protein levels, which were determined by the bicinchoninic acid (BCA) assay. The results were analyzed using GraphPad Prism 8.0.

### mtDNA quantification

To quantify mtDNA, 2500 embryos, 600 L2s and 40 L4s were lysed with 40 μL of worm PCR lysis buffer. DNA was released by heating the worms in a PCR cycler at 65 °C for 90 min and then 95 °C for 15 min. The worm lysate was diluted 50 times with nuclease-free water, and 5 μL of the sample was used as the template for RT-PCR. The ratio of the mitochondrial gene *nd-1* and the nuclear gene *act-3* expression levels was used to represent the relative levels of mtDNA per nuclear genome.

### Western blot analysis

Pellets of 100,000 eggs obtained by bleaching, 20,000 L2s, 5000 L4s and 2000 3-day-old adults were collected and snap-frozen with liquid nitrogen. All worm samples were preserved in a –80 °C freezer before use. For western blot assays, all samples were thawed on ice and lysed in RIPA-mid (Beyotime, P0013C) with sonication. To avoid dephosphorylation of the AAK-2 (AMPKα) protein, a phosphatase inhibitor was added to the samples before sonication. The worm lysates were then centrifuged at 16,000× *g* for 10 min at 4 °C, and the supernatant was collected for protein concentration determination with a BCA protein assay. Forty micrograms of total protein per well was loaded. The membranes were incubated with primary antibodies anti-pAMPK (Cell Signaling, 2535S, 1:1000, diluted with 2% BSA) and anti-tubulin (Abcam, ab6161, 1:3000, diluted with 2% BSA) and with HRP goat-anti-rabbit (Abclonal, AS014, 1:10,000, diluted with 5% milk), and HRP goat-anti-rat (Beyotime, A0192, 1:5000, diluted with 5% milk) secondary antibodies.

### DBL-1 secretion and TGFβ signaling activity test

For DBL-1 secretion measurement, a pair of coelomocytes near the pharynx were imaged with a Leica microscope with a 63× oil objective. For each pair of coelomocytes, the clearest focal plane was captured and analyzed in ImageJ to calculate an average fluorescence intensity value. The fluorescence intensity of the fluorescent patches inside coelomocytes was measured and normalized by subtracting background fluorescence.

For the TGFβ signaling activity test, fluorescence images of RAD-SMAD reporter animals were taken with a Leica microscope using a 40× objective. Each hypodermal nucleus fluorescence intensity was determined according to the average intensity of manually selected individual nuclei and analyzed by ImageJ. The fluorescence intensity for each hypodermal nucleus was measured, and the background fluorescence was subtracted.

### Statistical analysis

The statistical analyses were performed using GraphPad Prism 8.0. Statistical significance was calculated by unpaired *t*-test. The data were presented as the means ± SEM with indicated *P* values.

### Supplementary information

Supplementary information, Figure S1

Supplementary information, Figure S2

Supplementary information, Figure S3

Supplementary information, Figure S4

Supplementary information, Figure S5

Supplementary information, Figure S6

Supplementary information, Figure S7

Supplementary information, Figure S8

Supplementary information, Figure S9

Supplementary information, Figure S10

Supplementary information, Figure S11

Supplementary information, Figure S12

Supplementary information, Figure S13

Supplementary information, Table S1

## Data Availability

The UK Biobank data are available through formal application to the UK Biobank (http://www.ukbiobank.ac.uk). All materials or other data supporting points of this study are available from the corresponding authors upon reasonable request.
