# Supplementary material for: Maternal aging increases offspring adult body size via transmission of donut-shaped mitochondria
Source: Cell Res. 2023 Jul 27;33(11):821–34. doi: 10.1038/s41422-023-00854-8 (PMC10624822; doi:10.1038/s41422-023-00854-8)
Supplement: Supplementary file 1 — Supplementary information, Figure S1 [file 41422_2023_854_MOESM1_ESM.pdf]

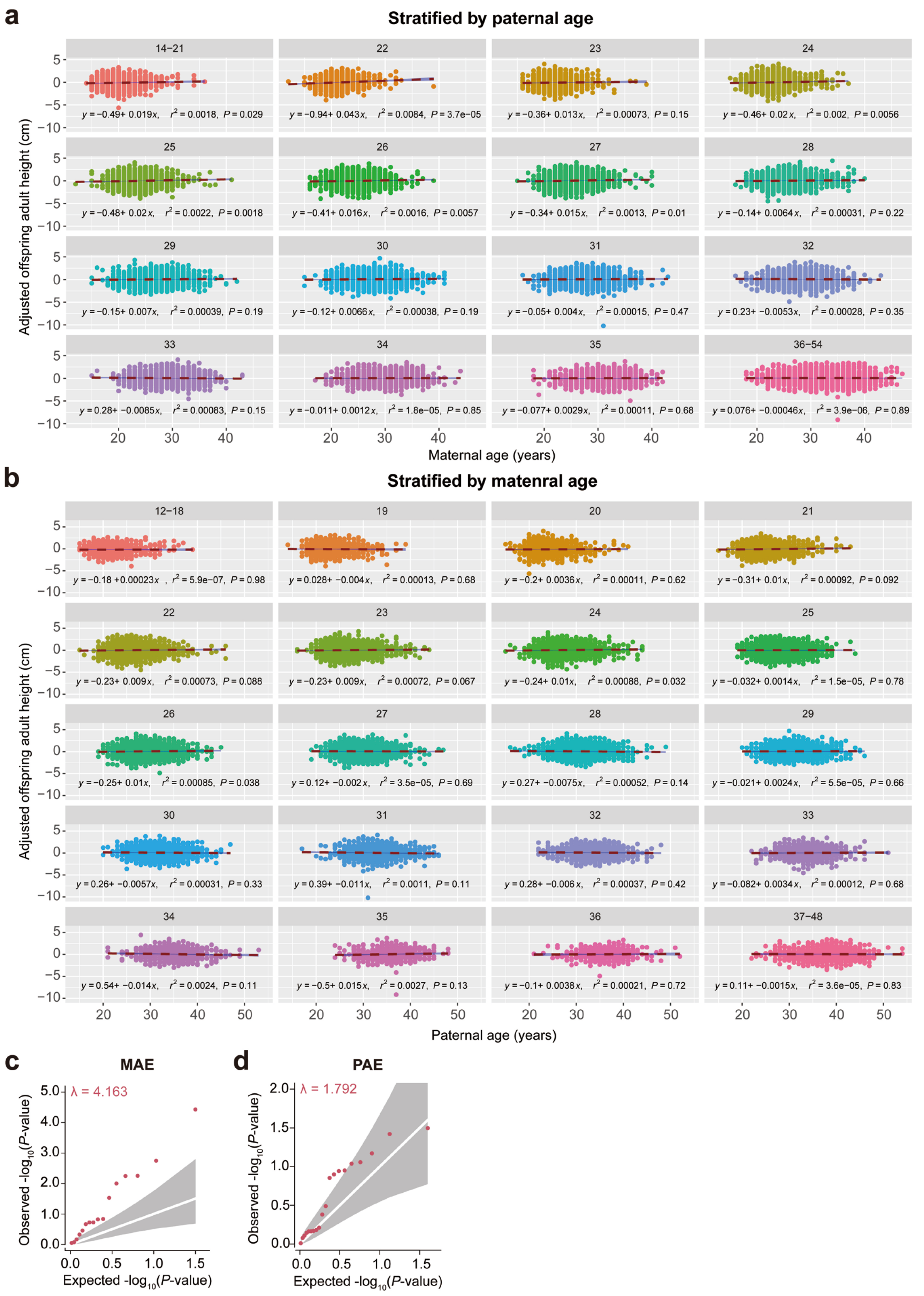

Fig. S1 Parental age-stratified analysis of the human data from the UKB. a, Relationship between maternal age and offspring adult height when paternal age was restricted to a particular value or a small range. Each grid corresponds to a paternal age value (or range), and each dotted line represents the regression slope. **b**, Relationship between paternal age and offspring adult height when the parent–offspring pairs were

stratified by maternal age. Each grid corresponds to a maternal age value (or range), and each dotted line represents the regression slope. **c**, Quantile–quantile plot of the association test between maternal age and offspring adult height across a range of paternal ages. **d**, Quantile–quantile plot of the association test between paternal age and offspring adult height across a range of maternal ages.
