# Supplementary material for: Maternal aging increases offspring adult body size via transmission of donut-shaped mitochondria
Source: Cell Res. 2023 Jul 27;33(11):821–34. doi: 10.1038/s41422-023-00854-8 (PMC10624822; doi:10.1038/s41422-023-00854-8)
Supplement: Supplementary file 2 — Supplementary information, Figure S2 [file 41422_2023_854_MOESM2_ESM.pdf]

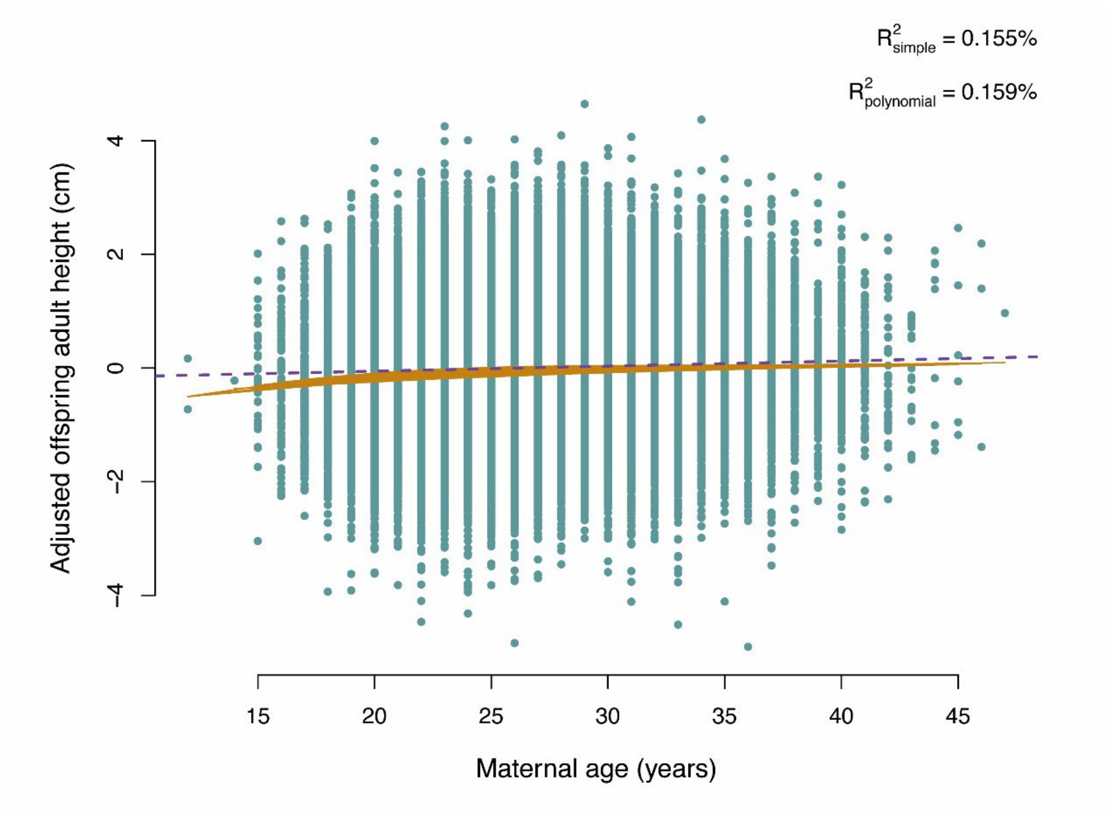

Fig. S2 Relationship between maternal age and offspring adult height in the UKB data analysis. The orange line represents the fitted polynomial regression curve, and the purple dashed line depicts the fitted simple regression curve.
