# Supplementary material for: Maternal aging increases offspring adult body size via transmission of donut-shaped mitochondria
Source: Cell Res. 2023 Jul 27;33(11):821–34. doi: 10.1038/s41422-023-00854-8 (PMC10624822; doi:10.1038/s41422-023-00854-8)
Supplement: Supplementary file 3 — Supplementary information, Figure S3 [file 41422_2023_854_MOESM3_ESM.pdf]

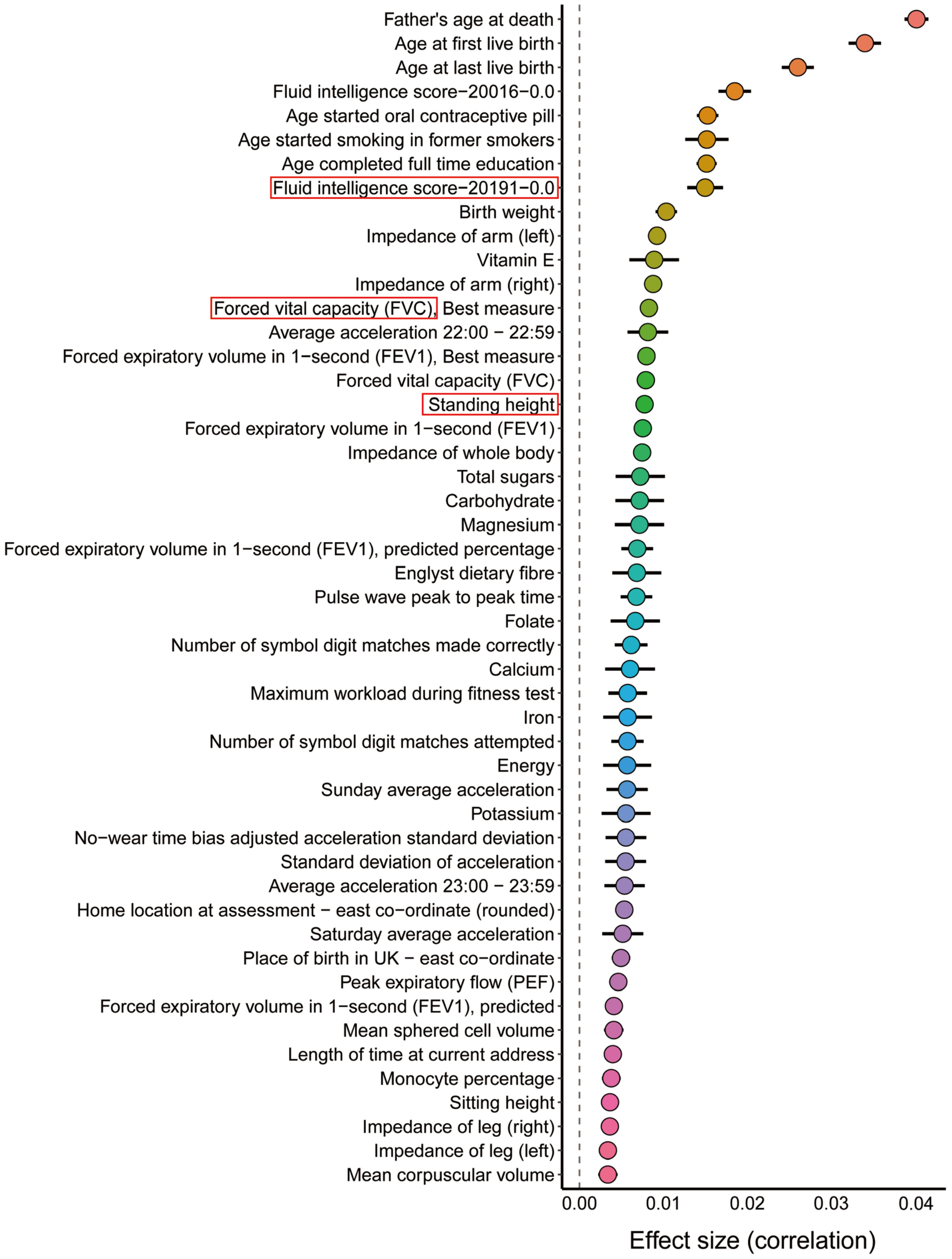

Father's age at death
Age at first live birth
Age at last live birth
Fluid intelligence score−20016−0.0
Age started oral contraceptive pill
Age started smoking in former smokers
Age completed full time education
Fluid intelligence score−20191−0.0
Birth weight
Impedance of arm (left)
Vitamin E
Impedance of arm (right)
Forced vital capacity (FVC), Best measure
Average acceleration 22:00 − 22:59
Forced expiratory volume in 1−second (FEV1), Best measure
Forced vital capacity (FVC)
Standing height
Forced expiratory volume in 1−second (FEV1)
Impedance of whole body
Total sugars
Carbohydrate
Magnesium
Forced expiratory volume in 1−second (FEV1), predicted percentage
Englyst dietary fibre
Pulse wave peak to peak time
Folate
Number of symbol digit matches made correctly
Calcium
Maximum workload during fitness test
Iron
Number of symbol digit matches attempted
Energy
Sunday average acceleration
Potassium
No−wear time bias adjusted acceleration standard deviation
Standard deviation of acceleration
Average acceleration 23:00 − 23:59
Home location at assessment − east co−ordinate (rounded)
Saturday average acceleration
Place of birth in UK − east co−ordinate
Peak expiratory flow (PEF)
Forced expiratory volume in 1−second (FEV1), predicted
Mean sphered cell volume
Length of time at current address
Monocyte percentage
Sitting height
Impedance of leg (right)
Impedance of leg (left)
Mean corpuscular volume
0.00
0.01
0.02
0.03
0.04
Effect size (correlation)

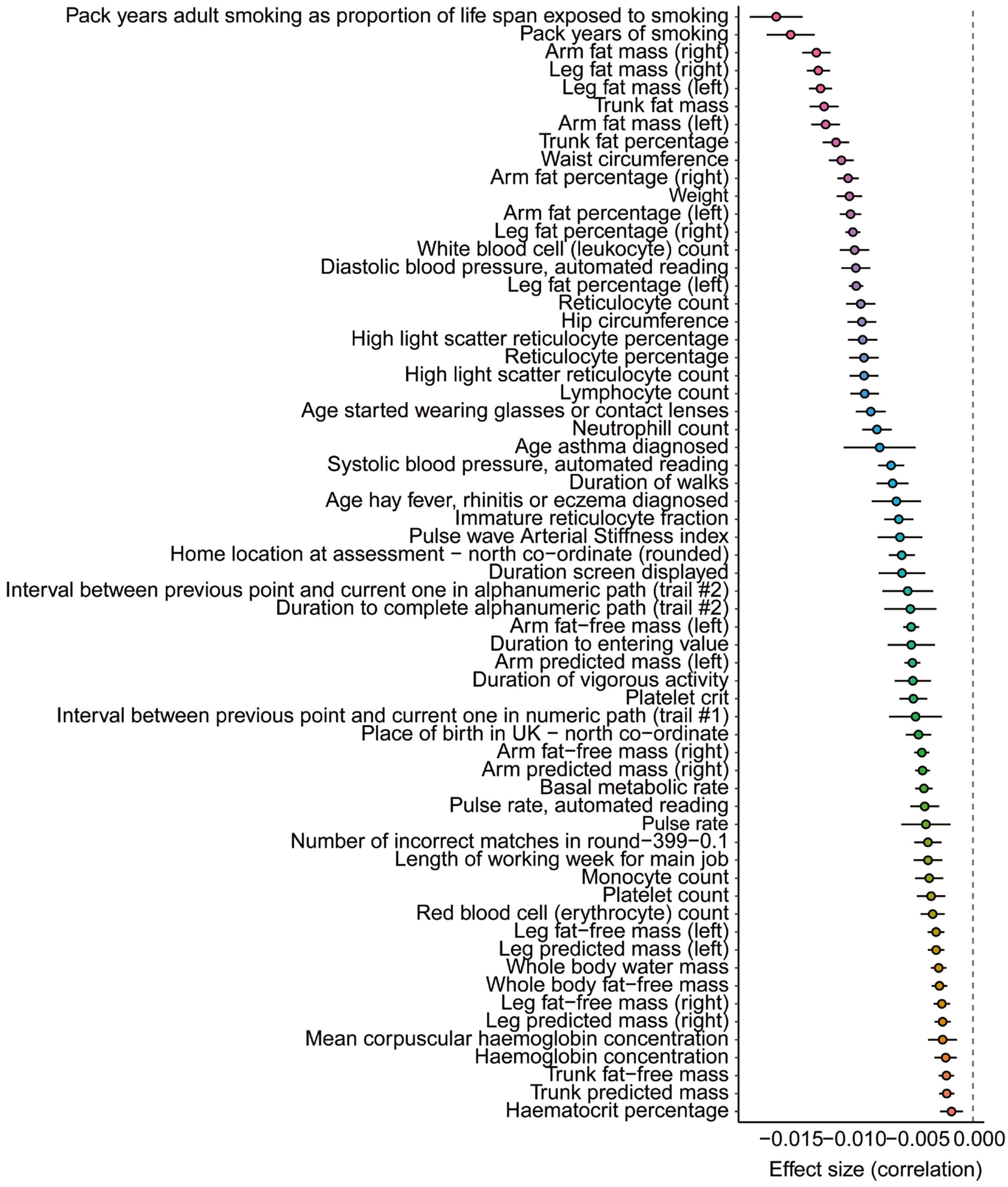

Fig. S3 Associations between maternal age and a host of phenotypes in the UKB data. Each error bar represents the 95% confidence interval around a correlation estimate.
