# Supplementary material for: Maternal aging increases offspring adult body size via transmission of donut-shaped mitochondria
Source: Cell Res. 2023 Jul 27;33(11):821–34. doi: 10.1038/s41422-023-00854-8 (PMC10624822; doi:10.1038/s41422-023-00854-8)
Supplement: Supplementary file 4 — Supplementary information, Figure S4 [file 41422_2023_854_MOESM4_ESM.pdf]

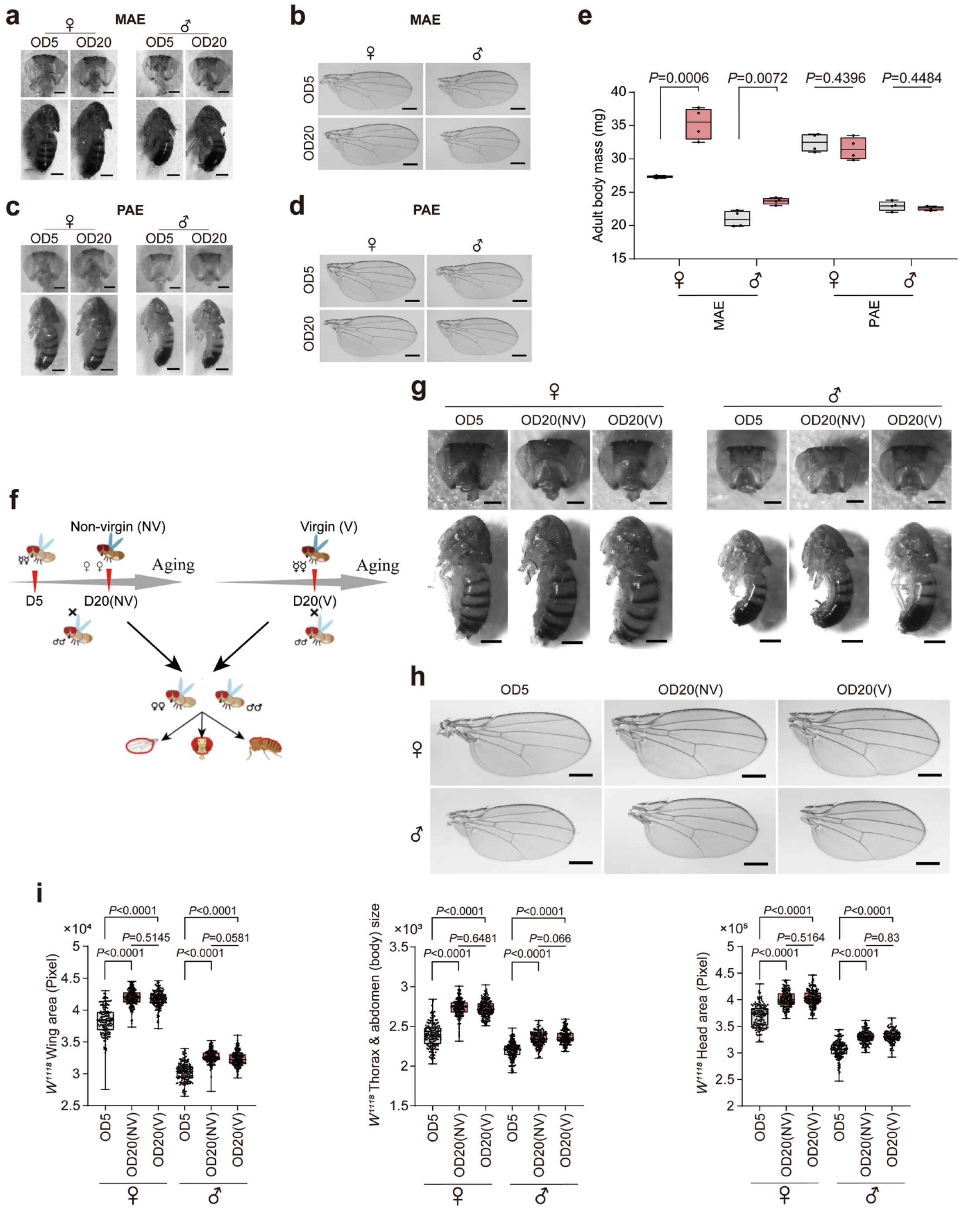

Fig. S4 MAE-mediated changes in adult flies. a, **b** Representative images of the head and body (**a**) and wings (**b**) of offspring with the indicated genotypes from young (OD5) and aged (OD20) mothers. **c**, **d** Representative images of the head and body (**c**) and wings (**d**) of offspring with the indicated genotypes from young (OD5) and aged (OD20) fathers. **e** Body mass changes by MAE or PAE in adult flies. Each dot represents the body mass of 30 flies. **f**–**i** Experimental scheme (**f**), representative images (**g**, **h**) and size measurements (**i**) of offspring flies born to virgin (V) and non-virgin (NV) mothers

for maternal parity test. The dots represent the fly numbers analyzed. Bars represent 200 µm (Head) or 400 µm (Body and wing). The data are presented as the mean ± SEM with box. Box plots in (**e**, **i**). In the box plots, the centerline is the median, the box range as the 25th–75th percentiles, and the whiskers indicating the minimum–maximum values. The box plots were analyzed by unpaired *t*-test. Biological replicates: 3 (**e**, **i**).
