# Supplementary material for: Maternal aging increases offspring adult body size via transmission of donut-shaped mitochondria
Source: Cell Res. 2023 Jul 27;33(11):821–34. doi: 10.1038/s41422-023-00854-8 (PMC10624822; doi:10.1038/s41422-023-00854-8)
Supplement: Supplementary file 5 — Supplementary information, Figure S5 [file 41422_2023_854_MOESM5_ESM.pdf]

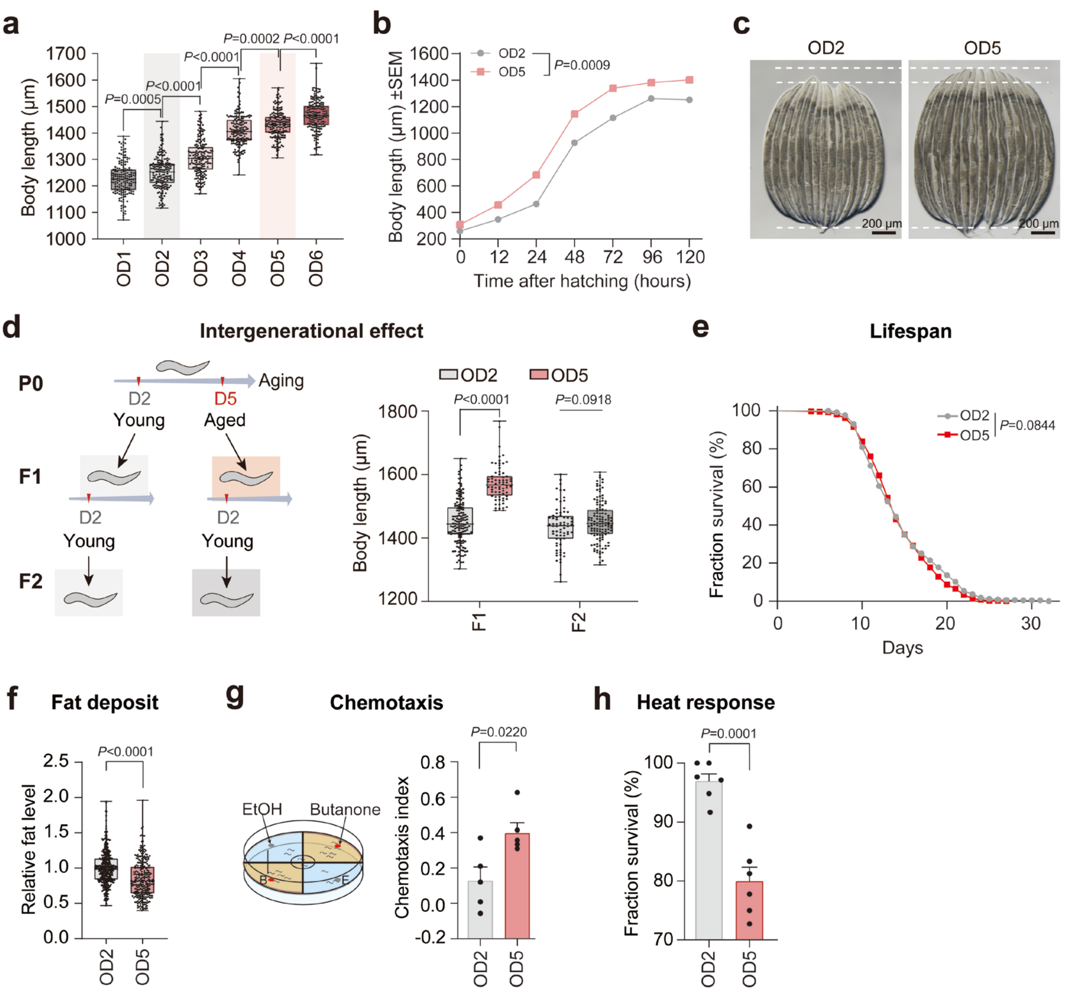

Fig. S5 MAE on offspring adult traits in *C. elegans*. **a** Adult body length of offspring born to mothers of different ages. **b** Body length comparisons between OD2 and OD5 animals during developmental processes. **c** Representative images of offspring of Day 2 (D2) mothers (OD2) and OD5 offspring. **d** Experimental design (left) and the obtained results (right) for transgenerational property exploration of MAEs. F1 offspring were produced by D2 (gray) and D5 (red) P0 mothers, while F2 offspring were produced by D2 F1 mothers. **e** Longevity assay for wild-type OD2 and OD5 offspring. **f** Relative fat level comparison between OD2 and OD5 animals. **g** Chemotaxis index comparison between OD2 and OD5 animals. **h** Heat response comparison between OD2 and OD5 animals. Dots in the box plots represent worm numbers, and dots in the bar plots represent biological replicates. The data are presented as the mean ± SEM with box plots in (**a**, **d**, **f**) or bar plots in (**g**, **h**). In the box plots, the centerline is the median, the box range shows the 25th–75th percentiles, and the whiskers indicate the minimum–maximum values. The box plots (**a**, **d**, **f**) and bar plots (**g**, **h**) were analyzed by unpaired

t-test. Biological replicates: 3 (**a**, **b**, **d**), 8 (**e**), 5 (**f**, **g**), 6 (**h**).
