# Supplementary material for: Maternal aging increases offspring adult body size via transmission of donut-shaped mitochondria
Source: Cell Res. 2023 Jul 27;33(11):821–34. doi: 10.1038/s41422-023-00854-8 (PMC10624822; doi:10.1038/s41422-023-00854-8)
Supplement: Supplementary file 6 — Supplementary information, Figure S6 [file 41422_2023_854_MOESM6_ESM.pdf]

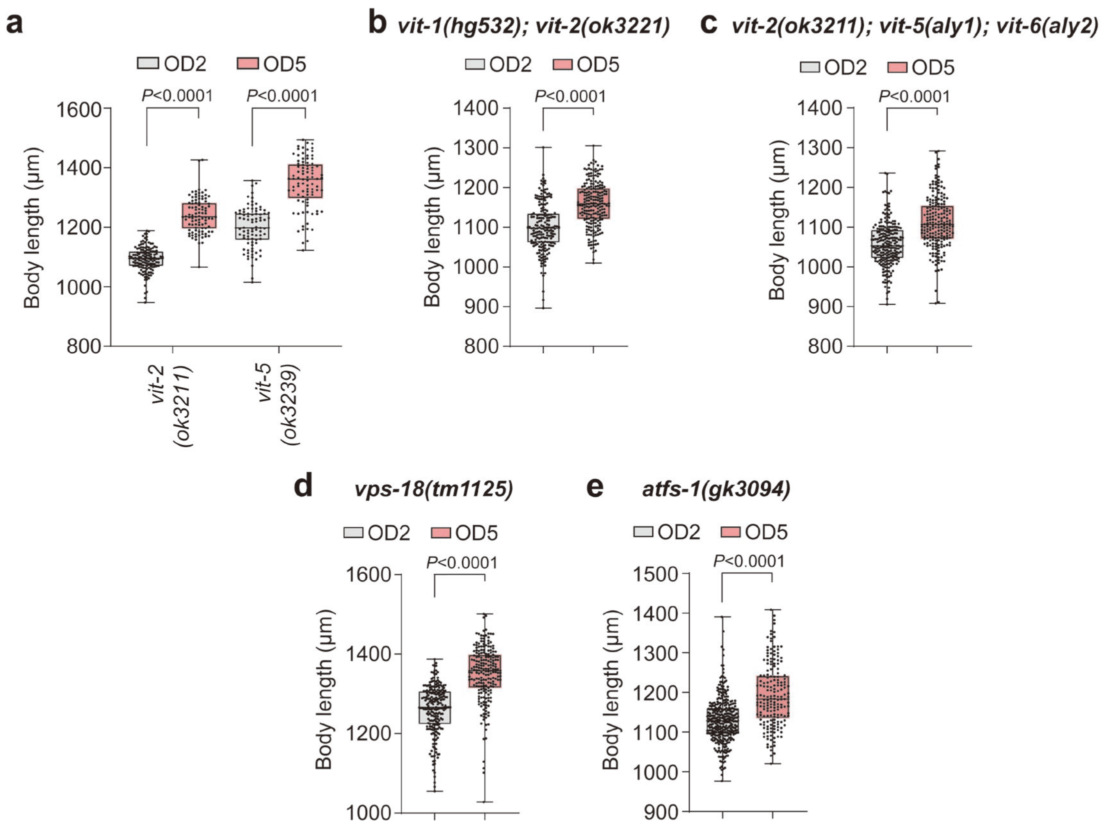

Fig. S6 The MAE-mediated offspring adult size change is independent of activities of vitellogenin, lysosome or mitochondrial UPR. a–e Body length comparisons between offspring of Day 2 (D2) mother (OD2) and OD5 animals with single, double or triple mutations in indicated vitellogenin (*vit*) (**a**–**c**), lysosomal (**d**) or UPR^{mt} (**e**) regulator genes. Dots in the box plots represent worm numbers. Box plots: the centerline is the median, the box range shows the 25th–75th percentiles, and the whiskers indicate the minimum–maximum values. The box plots were analyzed by unpaired *t*-test. Biological replicates: 3.
