# Supplementary material for: Maternal aging increases offspring adult body size via transmission of donut-shaped mitochondria
Source: Cell Res. 2023 Jul 27;33(11):821–34. doi: 10.1038/s41422-023-00854-8 (PMC10624822; doi:10.1038/s41422-023-00854-8)
Supplement: Supplementary file 7 — Supplementary information, Figure S7 [file 41422_2023_854_MOESM7_ESM.pdf]

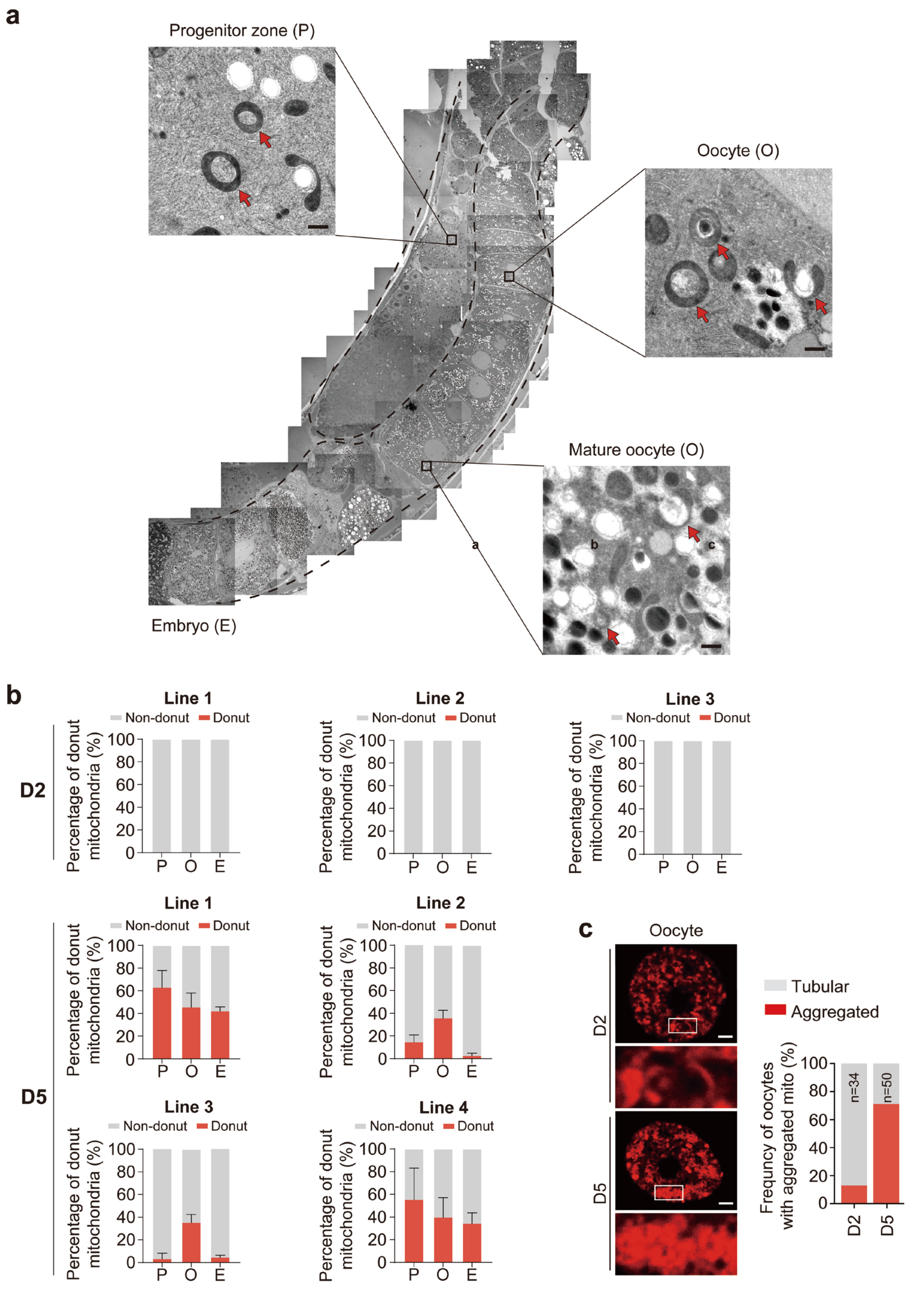

Fig. S7 Donut-shaped mitochondria were transmitted from a mother to oocytes. a, b Representative cross-sectional transmission electron microscopy (TEM) images (**a**) and the percentages of donut-shaped mitochondria (**b**) observed in the progenitor zones (P), oocytes (O) and embryos (E) in the germline of each imaged Day 2 (D2) and D5

animals. Each line represents a worm, and five images from each region were used for quantification. Red arrows indicate donut-shaped mitochondria. The scale bars represent 0.5 μm. **c** Confocal images (left) and the quantified ratios (right) of mitochondria stained by CNB in wild-type (WT) D2 and D5 oocytes. The scale bars represent 5 μm. The percentage of worms with aggregated mitochondria was shown in right bar plot.
