# Supplementary material for: Maternal aging increases offspring adult body size via transmission of donut-shaped mitochondria
Source: Cell Res. 2023 Jul 27;33(11):821–34. doi: 10.1038/s41422-023-00854-8 (PMC10624822; doi:10.1038/s41422-023-00854-8)
Supplement: Supplementary file 8 — Supplementary information, Figure S8 [file 41422_2023_854_MOESM8_ESM.pdf]

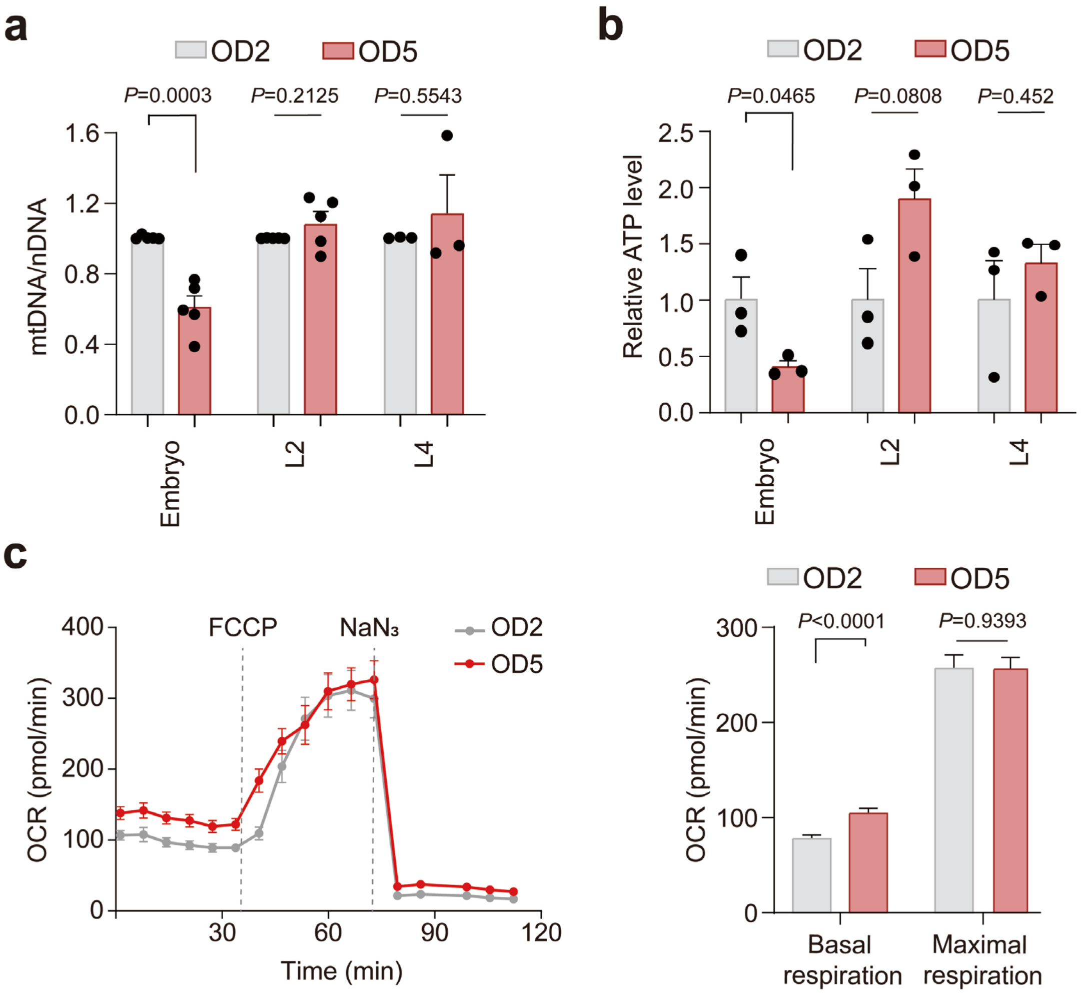

Fig. S8 Offspring mitochondrial mass and activity changes with maternal aging. **a**, **b**, Mitochondrial DNA (mtDNA) copy number (**a**) and relative ATP levels (**b**) of offspring of Day 2 (D2) mother (OD2) and OD5 animals in the embryonic, larval 2 (L2) and L4 stages. Dots in the bar plot represent biological replicates. **c**, The oxygen consumption rate test in OD2 and OD5 animals in L2 stages. The data are presented as the mean ± SEM in bar plots in (**a**-**c**). The data were analyzed by unpaired t test. Biological replicates: 3 (**a**-**c**).
