# Supplementary material for: Maternal aging increases offspring adult body size via transmission of donut-shaped mitochondria
Source: Cell Res. 2023 Jul 27;33(11):821–34. doi: 10.1038/s41422-023-00854-8 (PMC10624822; doi:10.1038/s41422-023-00854-8)
Supplement: Supplementary file 9 — Supplementary information, Figure S9 [file 41422_2023_854_MOESM9_ESM.pdf]

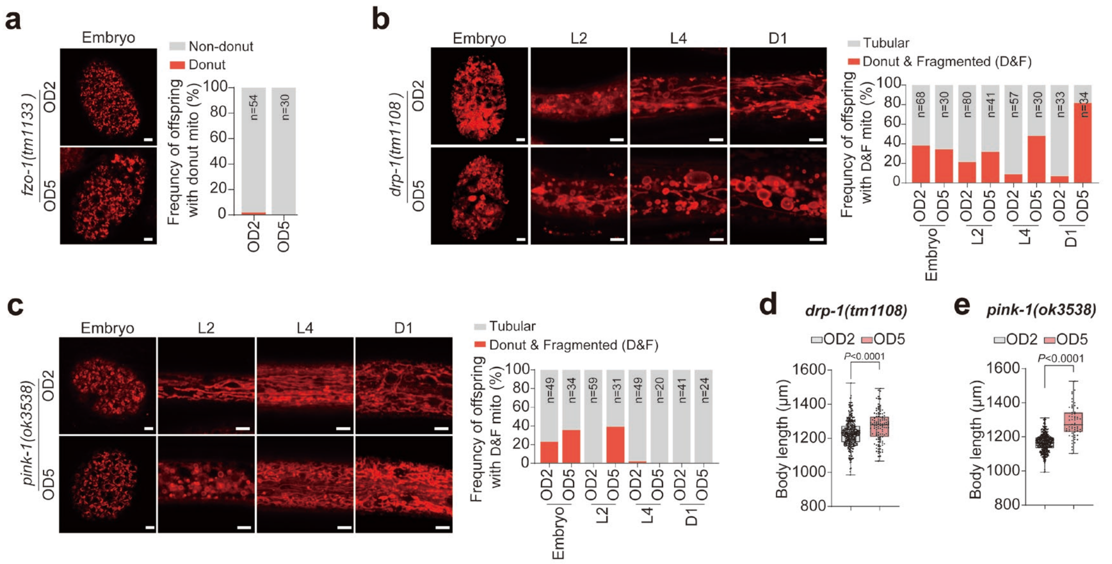

Fig. S9 Aggregation and rejuvenation of donut-shaped mitochondria and the MAE-mediated offspring adult size changes in the mutants with defects in mitochondrial dynamics. a–c Confocal images (left) and the quantified ratios (right) of mitochondria stained by CNB in mitochondrial fusion (*fzo-1*), fission (*drp-1*) or mitophagy (*pink-1*) mutant animals in embryonic or from embryonic to early adult stages. The scale bars represent 5 μm. Percentages of worms with donut-shaped mitochondria were shown in the right bar plot. **d**, **e** Adult body length comparisons between OD2 and OD5 animals born to *drp-1(tm1108)* or *pink-1(ok3538)* mutant mothers. Box plots in (**d**, **e**): the centerline is the median, the box range shows the 25th–75th percentiles, and the whiskers indicate the minimum-maximum values. The box plots were analyzed by unpaired *t*-test. Biological replicates: 3 (**d**, **e**).
