# Supplementary material for: Maternal aging increases offspring adult body size via transmission of donut-shaped mitochondria
Source: Cell Res. 2023 Jul 27;33(11):821–34. doi: 10.1038/s41422-023-00854-8 (PMC10624822; doi:10.1038/s41422-023-00854-8)
Supplement: Supplementary file 10 — Supplementary information, Figure S10 [file 41422_2023_854_MOESM10_ESM.pdf]

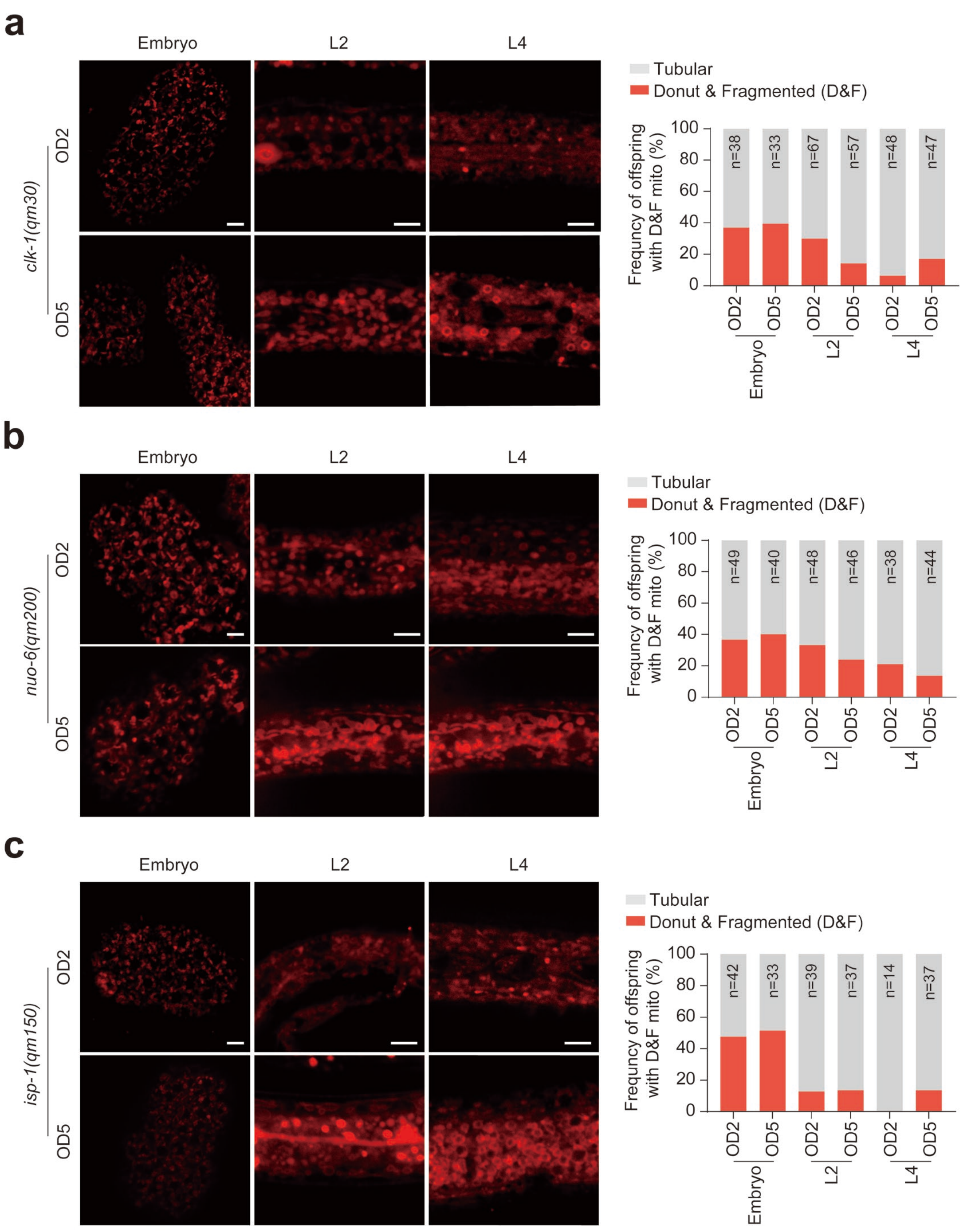

Fig. S10 Aggregation and rejuvenation of donut-shaped mitochondria in the mitochondrial mutant animals. a–c Confocal images (left) and the quantified ratios (right) of mitochondria stained by CNB in the mitochondrial mutants, including *clk-1(qm30)* (**a**), *nuo-6(qm200)* (**b**) and *isp-1(qm150)* (**c**), from embryonic to L4 stages. The scale bars represent 5 μm. Percentages of worms with donut-shaped mitochondria were shown in right bar plot.
