# Supplementary material for: Maternal aging increases offspring adult body size via transmission of donut-shaped mitochondria
Source: Cell Res. 2023 Jul 27;33(11):821–34. doi: 10.1038/s41422-023-00854-8 (PMC10624822; doi:10.1038/s41422-023-00854-8)
Supplement: Supplementary file 11 — Supplementary information, Figure S11 [file 41422_2023_854_MOESM11_ESM.pdf]

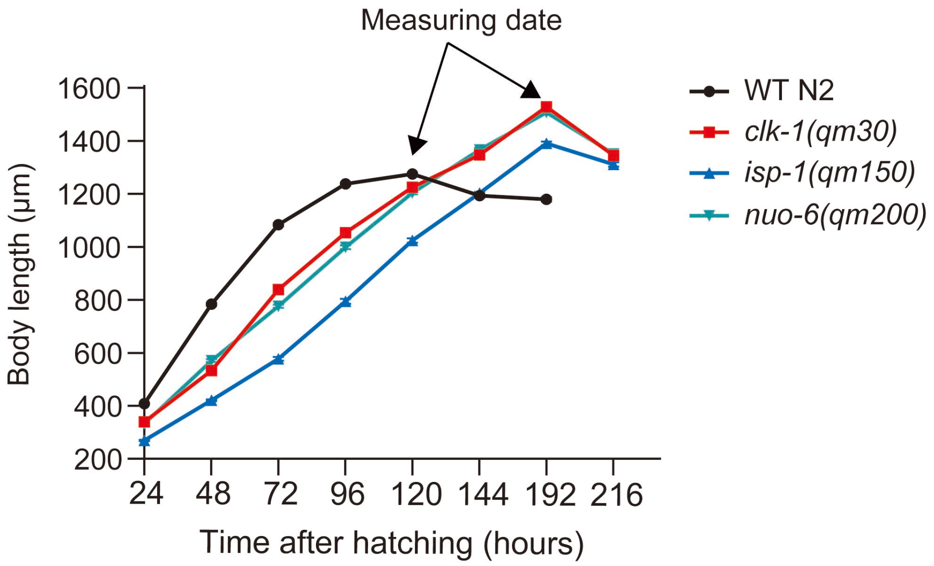

Fig. S11 Body length comparisons among WT and mitochondrial dysfunctional animals. Body length measurements in wild-type (WT) and the mitochondrial ETC mutant animals during their developmental processes.
