# Supplementary material for: Maternal aging increases offspring adult body size via transmission of donut-shaped mitochondria
Source: Cell Res. 2023 Jul 27;33(11):821–34. doi: 10.1038/s41422-023-00854-8 (PMC10624822; doi:10.1038/s41422-023-00854-8)
Supplement: Supplementary file 12 — Supplementary information, Figure S12 [file 41422_2023_854_MOESM12_ESM.pdf]

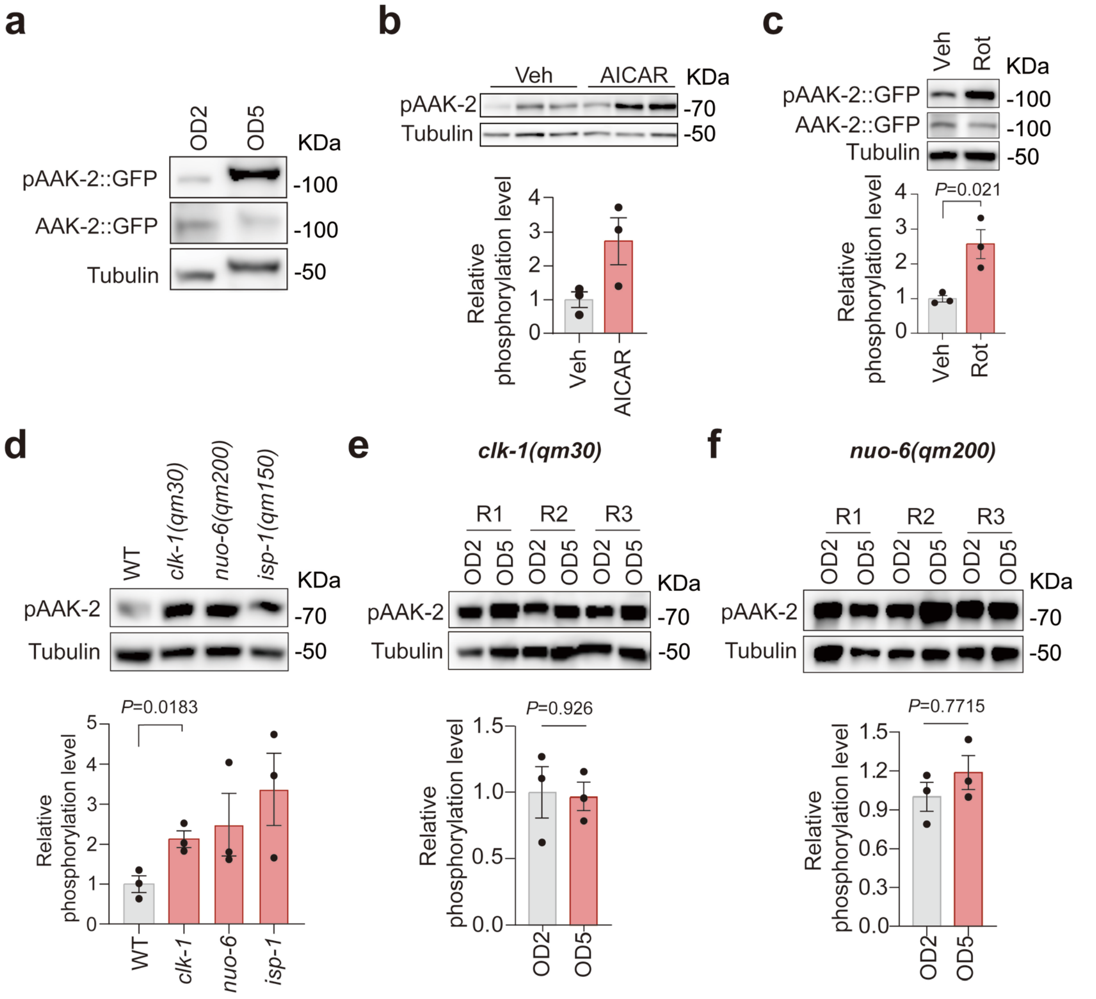

Fig. S12 The AMPK expression and activity test in animals treated with indicated compounds, MAE or mutations in mitochondrial genes. a Western blot analysis of the AAK-2 phosphorylation and total protein levels in OD2 and OD5 embryos carrying a GFP-knockin tag by CRISPR at the C-terminus of AAK-2. **b** Western blot analysis of AAK-2 phosphorylation levels in OD2 L2 animals treated with AICAR from eggs. **c** Western blot analysis of AAK-2 phosphorylation levels in OD2 embryos of rotenone treated mothers. **d**–**f** Western blot analysis of AAK-2 phosphorylation levels in mitochondrial mutant embryos, including *clk-1(qm30)*, *nuo-6(qm200)* and *isp-1(qm150)*. Representative immunoblots are displayed in the upper panel, and the quantification results are shown with bar plots in the lower panel. Dots in bar plots represent biological replicates. The bar plots were analyzed by unpaired *t*-test.
