# Supplementary material for: Maternal aging increases offspring adult body size via transmission of donut-shaped mitochondria
Source: Cell Res. 2023 Jul 27;33(11):821–34. doi: 10.1038/s41422-023-00854-8 (PMC10624822; doi:10.1038/s41422-023-00854-8)
Supplement: Supplementary file 13 — Supplementary information, Figure S13 [file 41422_2023_854_MOESM13_ESM.pdf]

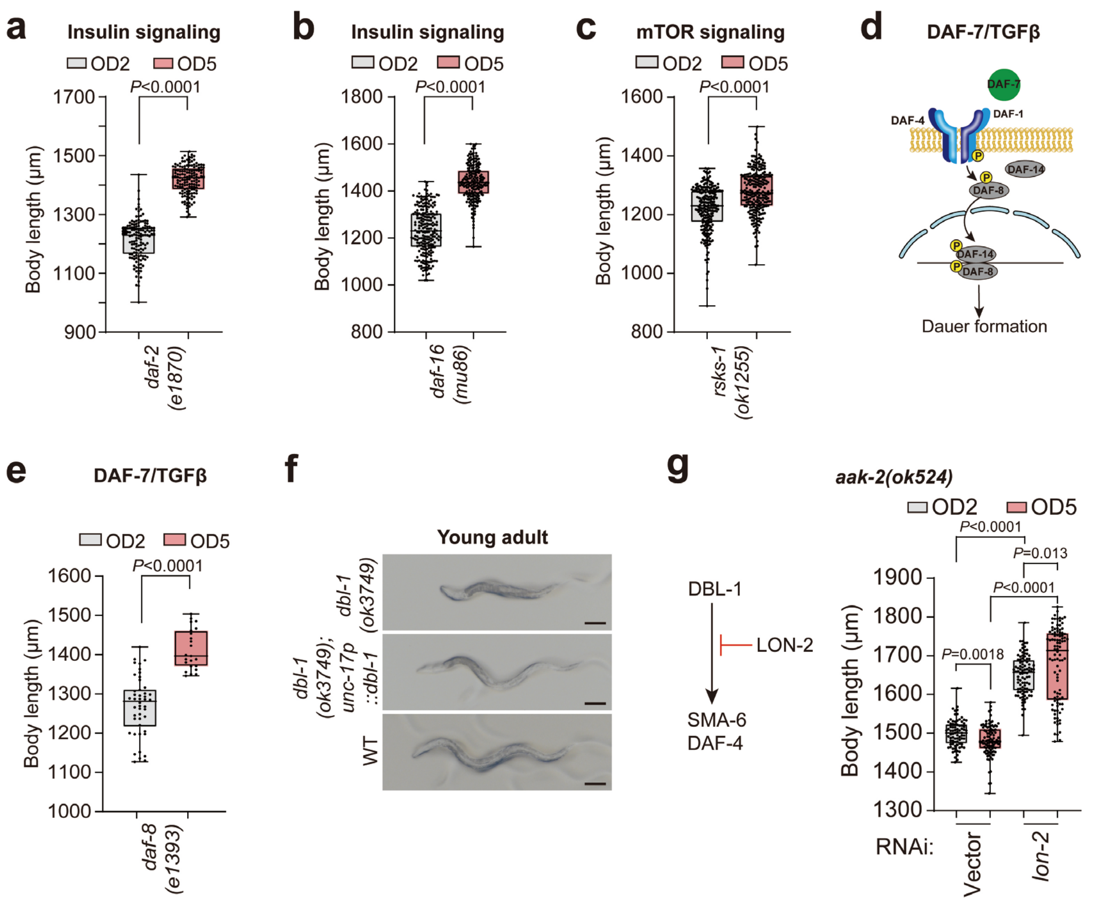


**Fig. S13 Exploration of AMPK downstream effectors in response to MAE. a, b** Adult body length comparison between offspring of OD2 and OD5 animals born to *daf-2(e1370)* (**a**) or *daf-16(mu86)* (**b**) mutant animals. **c** Adult body length comparisons of OD2 and OD5 offspring of *rsks-1(ok1255)* mutant animals. **d** *C. elegans* DAF-7/TGFβ pathway scheme. **e** Adult body length comparisons between OD2 and OD5 offspring of *daf-8(e1393)* mutant animals. **f** Representative images of *dbl-1(ok3749)* mutant animals with or without the neuron-specific rescue. The scale bars represent 100 μm. **g** Adult body length measurements in OD2 and OD5 *aak-2(ok524)* mutant animals treated by vector or *lon-2* RNAi. Dots in the box plots represent worm numbers. Box plots in (**a**, **b**, **c**, **e**, **g**): the centerline is the median, the box range shows the 25th–75th percentiles, and the whiskers indicate the minimum–maximum values. The box plots were analyzed by unpaired *t*-test. Biological replicates: 3 (**a**, **b**, **c**, **e**, **g**).
