# Supplementary material for: Maternal aging increases offspring adult body size via transmission of donut-shaped mitochondria
Source: Cell Res. 2023 Jul 27;33(11):821–34. doi: 10.1038/s41422-023-00854-8 (PMC10624822; doi:10.1038/s41422-023-00854-8)
Supplement: Supplementary file 14 — Supplementary information, Table S1 [file 41422_2023_854_MOESM14_ESM.pdf]

**Table S1 Summary of lifespan results**

| Condition | N | # | Mean LS | Std. error | 95% C.I. | Chi^2 | *P*-value | Bonferroni *P*-value |
|---|---|---|---|---|---|---|---|---|
| OD2 | 817 | 8 | 14.6 | 0.15 | 14.30 to 14.90 | 2.98 | 0.0844 | 0.0844 |
| OD5 | 642 | 8 | 14.33 | 0.16 | 14.02 to 14.64 | | | |
